# Transformable Neuropeptide Prodrug with Tumor Microenvironment Responsiveness for Tumor Growth and Metastasis Inhibition of Triple‐Negative Breast Cancer

**DOI:** 10.1002/advs.202300545

**Published:** 2023-05-05

**Authors:** Yi Cao, Xiaojiao Ge, Xueli Zhu, Yingying Han, Pin Wang, Ozioma Udochukwu Akakuru, Aiguo Wu, Juan Li

**Affiliations:** ^1^ Cixi Institute of Biomedical Engineering International Cooperation Base of Biomedical Materials Technology and Application CAS Key Laboratory of Magnetic Materials and Devices Zhejiang Engineering Research Center for Biomedical Materials Ningbo Institute of Materials Technology and Engineering Chinese Academy of Sciences 315201 Ningbo P. R. China; ^2^ University of Chinese Academy of Sciences 100049 Beijing P. R. China

**Keywords:** mitochondria targetability, neuropeptide, transformable prodrug, triple‐negative breast cancer, tumor growth and metastasis

## Abstract

Triple‐negative breast cancer (TNBC) has the worst prognosis among all breast cancer subtypes due to lack of specific target sites and effective treatments. Herein, a transformable prodrug (DOX‐P18) based on neuropeptide Y analogue with tumor microenvironment responsiveness is developed for TNBC treatment. The prodrug DOX‐P18 can achieve reversible morphological transformation between monomers and nanoparticles through the manipulation of protonation degree in different environments. It can self‐assemble into nanoparticles to enhance the circulation stability and drug delivery efficiency in the physiological environment while transforming from nanoparticles to monomers and being endocytosed into the breast cancer cells in the acidic tumor microenvironment. Further, the DOX‐P18 can precisely be enriched in the mitochondria, and efficiently activated by matrix metalloproteinases. Then, the cytotoxic fragment (DOX‐P3) can subsequently be diffused into the nucleus, generating a sustained cell toxicity effect. In the meanwhile, the hydrolysate residue P15 can assemble into nanofibers to construct nest‐like barriers for the metastasis inhibition of cancer cells. After intravenous injection, the transformable prodrug DOX‐P18 demonstrated superior tumor growth and metastasis suppression with much better biocompatibility and improved biodistribution compared to free DOX. As a novel tumor microenvironment‐responsive transformable prodrug with diversified biological functions, DOX‐P18 shows great potential in smart chemotherapeutics discovery for TBNC.

## Graphical abstract

Transformable neuropeptide prodrug DOX‐P18 provides responsive morphology transformation and drug release in the extracellular matrix and mitochondria, which can inhibit tumor growth and metastasis in a triple‐negative breast cancer‐bearing mouse model.

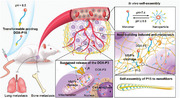

## 1 Introduction

Triple‐negative breast cancer (TNBC), the most aggressive breast cancer subtype, has become a great threat to women's health worldwide.^[^ ^1^ ^]^ Until now, chemotherapy is still the major established systemic treatment for patients with TNBC.^[^ ^2^ ^]^ However, the limited efficacy of traditional treatment persists, due to the lack of targeted therapies and high heterogeneity.^[^ ^3^ ^]^ Therefore, a major effort has been fostered to discover novel targets to treat patients with these aggressive cancers, such as BRCA1/BRCA2, androgen receptor, PI3K/AKT/mTOR, TROP2, PD‐1/PD‐L1,^[^ ^4^ ^]^ and numerous medications based on the above targets have been researched and clinically tested for treating TNBC.^[^ ^5^ ^]^ However, these trials have not yielded efficient clinical benefits.^[^ ^6^ ^]^ Thus, it is urgent to develop highly specific target site‐based personalized precision diagnosis and therapy for these patients.

Smart peptide‐based prodrugs as a new strategy for functional drug development with high specificity, selectivity, and flexibility are being actively explored.^[^ ^7^ ^]^ However, low circulation stability, limited tumor penetration, and less subcellular modulation of the peptides still hinder their further application in biological systems.^[^ ^8^ ^]^ As the most versatile motifs offer a much more functional and structural diversity, a peptide‐based morphological transformation strategy has been developed to overcome the shortcomings, such as short half‐life or low bioavailability, providing a more versatile pharmacokinetic optimization platform.^[^ ^9^ ^]^ Therefore, novel transformable prodrugs are needed to revolutionize peptide‐based drug discovery, leading to unlimited therapeutic possibilities.^[^ ^10^ ^]^

Neuropeptides are a class of bioactive peptide molecules with regulatory functions released by the central and peripheral neurological systems.^[^ ^11^ ^]^ More evidence has been proposed for the potential role of neuropeptides in the differentiation, proliferation, and metabolism of cancer cells, such as neurotensin, neuropeptide Y, and bombesin.^[^ ^12^ ^]^ Among them, neuropeptide Y (NPY) and its Y_1_ subtype receptors (Y_1_R) have been reported to be related to cancers,^[^ ^13^ ^]^ such as human breast carcinomas (85%) and breast cancer‐derived metastases (100%).^[^ ^14^ ^]^ The high and specific expression of Y_1_R in situ and in metastatic breast cancers positions specific NPY analogues as great potential agents for targeted imaging and therapy of TNBC.^[^ ^15^ ^]^ In previous work, we have focused on the specific targeting mechanism of NPY analogues to tumors and physiological barriers, revealing novel binding sites between novel NPY analogues and their subtype receptors,^[^ ^16^ ^]^ and developing a series of novel NPY analogues‐based nanosystems for visualized therapy of Y_1_R overexpressed tumors.^[^ ^17^ ^]^ However, the transformable prodrugs based on NPY analogues have not yet been developed until now, and the interactions between the transformable prodrugs and the physiological and pathological systems in vitro and in vivo still need to be extensively studied.

Here, we report the design, synthesis, and evaluation of a novel transformable prodrug with diversified biological functions, designated as DOX‐P18, whereby DOX is conjugated to a novel NPY analogue via matrix metalloproteins (MMPs) cleavable linker PLGVRG peptide. This transformable prodrug DOX‐P18 can assemble into nanoparticles in a physiological environment and disintegrate into monomers in an acidic tumor environment. Once exposed to MMPs in the tumor microenvironment, prodrug DOX‐P18 is activated to efficiently release the cytotoxic agent DOX‐P3, and the residue P15 self‐assembly in situ to form nanofibers in the tumor microenvironment. In this way, cancer cells are encircled to perish within the constructed barrier, which helps to enhance the retention of DOX‐P3 and suppress tumor growth and metastasis. Notably, better biocompatibility and improved biodistribution are demonstrated compared to free DOX in vivo. As a novel tumor microenvironment‐responsive transformable prodrug with diversified biological functions, DOX‐P18 showed great potential in chemotherapeutics discovery and efficacious tumor therapy for TBNC.

## 2 Results and Discussion

### 2.1 Design, Synthesis, and Characterization of the Transformable Prodrug

**Scheme 1:**
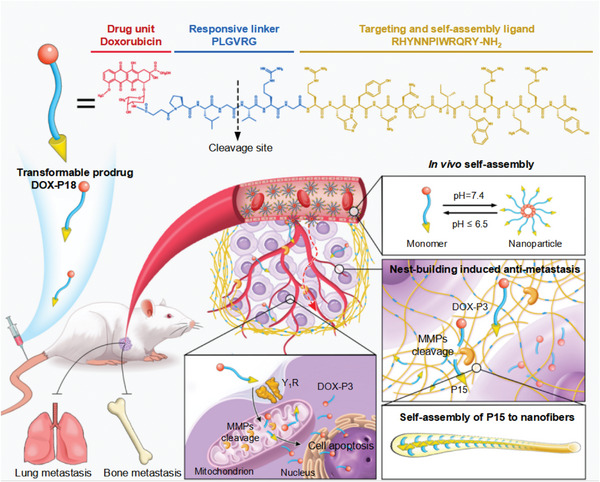
Molecular structure of neuropeptide Y analogue prodrug DOX‐P18, and the schematic representation of responsive morphology transformation and drug release of DOX‐P18 in the extracellular matrix and mitochondria for inhibiting tumor growth and metastasis on triple‐negative breast cancer‐bearing mouse model.

Transformable prodrug DOX‐P18 was designed by connecting peptide NH_2_‐PLGVRGRHYNNPIWRQRY‐CONH_2_ (abbreviated as P18) with anticancer drug doxorubicin (DOX) through succinic anhydride (Succ). Peptide NH_2_‐RHYNNPIWRQRY‐CONH_2_ ([Asn^28^, Pro^30^, Trp^32^]‐NPY (25‐36)), abbreviated as P12) was used as a target ligand for Y_1_R over‐expressed TNBC, and pentapeptide PLGVRG served as an MMPs responsive unit since MMPs is markedly a protease upregulated in metastatic malignant tumor. The structure and transform mechanism of DOX‐P18 are shown in **Scheme** **1**. Monodispersed DOX‐P18 in weak acidic buffers (pH ≤ 6.5) would assemble into nanoparticles while switching to a weak alkaline physiological environment (pH 7.4). Upon exposure to MMPs in a tumor environment, the specific cleavage of MMPs substrate peptide PLGVRG would induce prodrug DOX‐P18 into DOX‐P3. In this way, DOX‐P3 will be released as a much shorter peptide drug conjugate to help pass through the nuclear envelope and kill the cancer cells. While the residue P15 would form nanofibers to help the inhibition of tumor metastasis in triple‐negative breast cancer.

**Figure 1:**
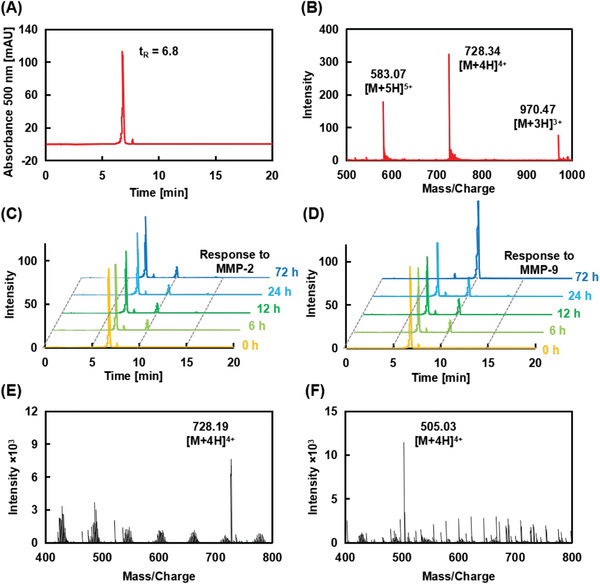
Characterization of DOX‐P18. A) Analytical HPLC of DOX‐P18 using an InfinityLab Poroshell 120 EC‐C18 column with a linear gradient of 5% to 95% (*v/v*) eluent B in eluent A in 20 min. B) ESI (+) mass spectra of DOX‐P18 displaying multiple charged molecular ions. C,D) HPLC analysis of DOX‐P18 (1 mg mL^−1^) after 6, 12, 24, and 72 h incubation with 1 µg mL^−1^ MMP‐2 or MMP‐9 at 37 °C using an InfinityLab Poroshell 120 EC‐C18 column with a linear gradient of 5% to 95% (*v/v*) eluent B in eluent A in 20 min. E,F) ESI (+) mass spectra of DOX‐P18 before and 72 h after incubation with MMP‐9.

The synthetic routes to DOX‐P18 and DOX‐P3 are illustrated in Scheme S1, Supporting Information. Peptide P18 and tripeptide NH_2_‐PLG‐COOH (abbreviated as P3) were prepared exclusively through the Fmoc‐based solid‐phase peptide synthesis. Briefly, P18 and P3 were obtained by conjugation of amino acids using N, N’‐diisopropylcarbodiimide (DIC) and 1‐hydroxybenzotriazole (HOBt). P18 linked with Succ was prepared with p‐nitrophenol and DIC activation. After full cleavage from resin, peptides were purified by reversed‐phase (RP)‐HPLC. Finally, DOX‐P18 and DOX‐P3 were built up by coupling the free amino group of DOX·HCl with 4‐methylmorpholine (NMM). The results of analytical HPLC and ESI mass spectrometry are exemplarily shown in **Figure** **1**. The analysis confirmed a purity above 95% for DOX‐P18 (Figure 1A) and DOX‐P3 (Figure S1A, Supporting Information). Representative MS spectra were depicted in Figure 1B and Figure S1B, Supporting Information to demonstrate the successful synthesis of DOX‐P18 and DOX‐P3. Spectral characteristics of DOX‐P18 shown in Figure S2A,B, Supporting Information revealed maximum light absorption at 500 nm, displaying a 10 nm red shift compared with the 490 nm for DOX, and the intensities of the maximum absorption were as strong as free DOX (Figure S2C,D, Supporting Information). However, fluorescence intensities of DOX‐P18 with consistent DOX concentration were drastically reduced to about five times lower than free DOX (Figure S3, Supporting Information), which may due to the connection with the carboxyl group of Succ.

### 2.2 Tumor Microenvironment Responsiveness of the Transformable Prodrug

Enzymatic responsiveness of transformable prodrug DOX‐P18 in the presence of matrix metalloproteinase‐2 (MMP‐2) or matrix metalloproteinase‐9 (MMP‐9) was validated by HPLC and MS in Figure 1. The HPLC results clearly showed that DOX‐P18 was efficiently cleaved by MMP‐2 (Figure 1C) or MMP‐9 (Figure 1D) to generate a new substance at 10.15 min. The enzymatic reaction rate monitored at 500 nm suggested that the PLGVRG segment in DOX‐P18 was more sensitive to MMP‐9 than MMP‐2. The newly produced substance was identified with MS as DOX‐P3 (Figure 1E,F). The recovery of DOX fluorescence intensity was also observed with the development of enzymatic reaction in Figure S4A, Supporting Information. The cleavage efficiency of MMP‐9 to DOX‐P18 analyzed by fluorescence intensity was about 10 times to MMP‐2 (Figure S4B, Supporting Information). All the above data proved the effective and site‐specific cleavage of DOX‐P18 in MMPs.

**Figure 2:**
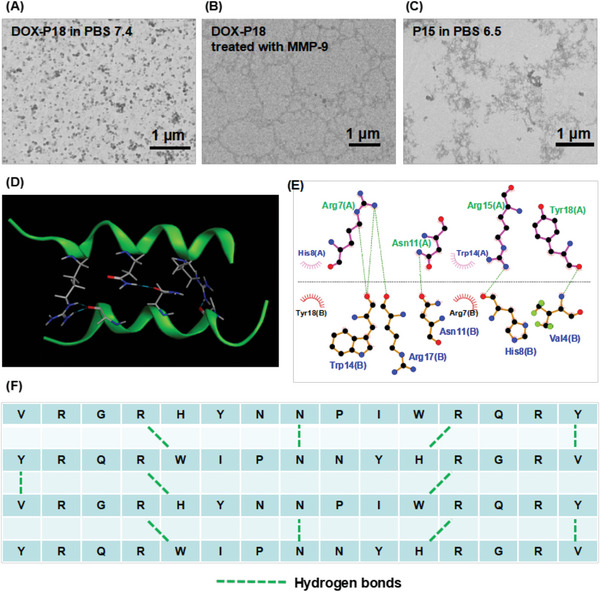
Study on morphology transformation of DOX‐P18. A–C) TEM images of DOX‐P18 in PBS 7.4, DOX‐P18 treated with 0.1 µg mL^−1^ MMP‐9 and P15 in PBS 6.5. D,E) Schematic representation of hydrogen‐bonding interactions between the dimer interface residues of P15 monomers. F) Schematic overview of sequence‐based interactions between P15 monomers.

To investigate the morphological transformation of the developed prodrug in an acidic tumor microenvironment, the particle size distribution of DOX‐P18 in PBS buffer with different pH (7.4, 6.5, 6.0, and 5.0) was measured by dynamic light scattering (DLS) (Table S1, Supporting Information). The results indicated that nanoparticles could only be detected in PBS buffer with pH 7.4 while remaining monomolecular dispersion in neutral and weakly acidic media (pH ≤ 6.5). Results combined with transmission electron microscope (TEM) imaging in **Figure** **2**A,B revealed the following morphological transformation of DOX‐P18 from monomolecular to nanofibers in tumor environment‐mimicked buffer. The fibroid aggregations of P15 in PBS with pH 6.5 was observed in Figure 2C, which hinted at the formation of nanofibers. Further, hydrogen‐bonding interactions between the dimer of P15 peptide were discovered by protein‐protein docking in Schrodinger software (Figure 2D,E). The tendency to generate nanofibers through these parallel interactions transmitted between intermolecular hydrogen bonds in P15 was shown in Figure 2F.

### 2.3 Cellular Uptake and Cytotoxicity Evaluation

The expression of MMPs and Y_1_R levels was confirmed in human mammary epithelial cell line MCF‐10A, human breast cancer cell lines MCF‐7, and MDA‐MB‐231 by immunocytochemistry and Western blot analysis. As shown in Figure S5, Supporting Information, the cell nuclei were stained with DAPI (blue) and detected fluorescence signal intensity (green) of protein labeled by Alexa Fluor 488 reflected the expression levels. MMP‐2 protein was highly expressed in low metastatic MCF‐7 cells, while MMP‐9 and Y_1_R proteins were obviously overexpressed in high metastatic MDA‐MB‐231 cells. The results of Western Blot in Figure S6, Supporting Information showed that the MMP‐2 expression levels were significantly higher in MCF‐7 cells, while the expression of MMP‐9 protein was positive in all three kinds of cell lines. Presumably, MMP‐9 played a leading role in the MMPs‐responsive cleavage of DOX‐P18 in MDA‐MB‐231 cells. These results also suggested the overexpression of Y_1_R in MDA‐MB‐231 cell lines, qualifying Y_1_R as a possible target for the NPY‐based transformable prodrug discovery.

To clarify the cellular uptake mechanism of transformable prodrug DOX‐P18, a flow cytometry assay was carried out to test the mean fluorescence intensity (MFI) of MDA‐MB‐231 cells under different treatments. The results in Figure S7, Supporting Information showed that the uptake of DOX‐P18 into MDA‐MB‐231 cells was strongly suppressed at 4 °C, implying that active processes such as endocytosis were involved in the internalization of DOX‐P18. To elucidate the contributions of various endocytosis pathways during the uptake of DOX‐P18, MFI of MDA‐MB‐231 cells was quantitatively investigated at 4 °C, lysosomotropic agent chloroquine (CQ, 100 µM), Y_1_R antagonist (BIBP3304, 20 µM), chlorpromazine (CPZ, 30 µM), amiloride (50 µM), and methyl‐*β*‐cyclodextrin (M*β*CD, 5 mM) treatment for 1 h at 37 °C. The results provided further evidence that DOX‐P18 at 40 µg mL^−1^ was taken up by cells through one or more endocytosis pathways including Y_1_R‐mediated endocytosis (20%), caveolin‐mediated endocytosis (14%), and micropinocytosis (27%).

**Figure 3:**
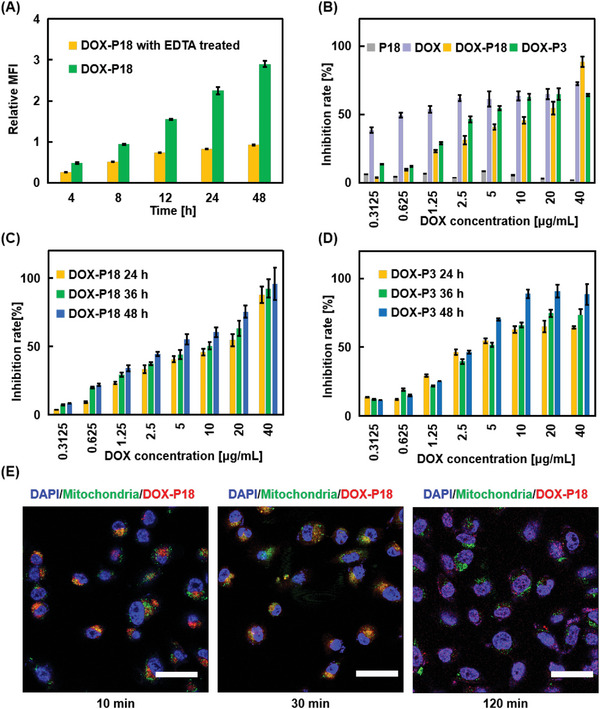
Cellular uptake and cytotoxicity evaluation of DOX‐P18. A) Flow cytometry analysis of DOX‐P18 incubated with MDA‐MB‐231 cells for 4, 8, 12, 24, and 48 h, with or without 5 mM EDTA treatment. Data are presented as means ± SD (*n* = 3). B) Cytotoxicity of P18, DOX, DOX‐P18, and DOX‐P3 in MDA‐MB‐231 cells after 24 h incubation. Data are presented as means ± SD (*n* = 3). C,D) Cell inhibition of DOX‐P18 and DOX‐P3 on MDA‐MB‐231 cells after 24, 36, and 48 h incubation. Data are presented as means ± SD (*n* = 3). E) Colocalization of DOX‐P18 (red) and DAPI (blue) or mitochondria tracker green (green) after 10, 30, and 120 min cultured with MDA‐MB‐231 cells. Scale bar: 25 µm.

Since DOX‐P18 could be efficiently cleaved in MMPs, an effective intracellular MMPs response of DOX‐P18 is an important prerequisite for prodrugs to achieve a better therapeutic effect. Therefore, a flow cytometry test was carried out to study the cellular drug release. The results in **Figure** **3**A showed that the fluorescence intensity in MDA‐MB‐231 cells reached saturation point at 48 h upon treatment of EDTA to inhibit the activity of MMPs. Unlike this trend, the fluorescence intensity in the DOX‐P18 group continuously increased even at 48 h, getting almost three times higher than that of the EDTA‐treated group, which revealed a constant drug release in the cells.

To distinguish the sustained release of DOX‐P18 induced by MMPs on different cell lines, DOX‐P18R was prepared as a non‐cleavable prodrug to clarify the natural degradation in the cell culture medium, in which random sequence PLGGRV replaced PLGVRG segment. The inhibition rate of DOX, DOX‐P18R, DOX‐P18, and DOX‐P3 on different cell lines upon 24 h co‐incubation was tested using a standard CCK‐8 cell viability assay (Figures S8–S10, Supporting Information). Mean IC_50_ values for cytotoxicity of DOX, DOX‐P18R, DOX‐P18, and DOX‐P3 were further obtained from the FORECAST function in Microsoft Excel (Table S2, Supporting Information). DOX‐P18R showed particularly high IC_50_ values on different cell lines, revealing a poor developability for chemotherapeutic drugs, which indicated that cleavable linkers were valuable approaches to activate prodrugs or release active compounds. IC_50_ values of DOX‐P18 on MCF‐10A and MCF‐7 cells were higher than those on MDA‐MB‐231 cells, which may be caused by different expression levels of MMPs. As an oligopeptide‐modified derivative of doxorubicin, DOX‐P3 acted as a mild toxophore compared to free DOX on three cell lines. Ulteriorly, the cytotoxicity of P18, DOX, DOX‐P18, and DOX‐P3 against MDA‐MB‐231 cells was systematically investigated (Figure 3B–D). No obvious cytotoxicity was detected with P18 to MDA‐MB‐231 cells even at a high concentration of 40 µg mL^−1^. Different degrees of cell inhibition were exhibited on DOX, DOX‐P18, and DOX‐P3, and their IC_50_ values were listed in Table S3, Supporting Information. As a powerful anticancer drug, DOX demonstrated the strongest cytotoxicity with an IC_50_ value of 1 µg mL^−1^ after 24 h co‐incubation with MDA‐MB‐231 cells, which was consistent with previous reports.^[^ ^18^ ^]^ However, the tested IC_50_ value of DOX‐P18 was about seven times higher (7.6 µg mL^−1^) than that of DOX, which pointed to the significantly improved safety of this NPY analogue‐based prodrugs. Meanwhile, as the effective unit of the prodrug, DOX‐P3 showed much stronger cell inhibition than DOX‐P18 (5.6 µg mL^−1^). The IC_50_ values of DOX‐P18 and DOX‐P3 were further reduced with extended incubation time, which may have resulted from continuous MMPs‐responsive cleavage.

To investigate the intracellular localization of DOX‐P18, confocal laser scanning microscopy (CLSM) was used for observation. As displayed in Figures S11 and S12, Supporting Information, few signals of the internalized DOX‐P18 were co‐localized within endosome or lysosome. In contrast, a high overlap of DOX‐P18 with the mitochondria tracker was found in Figure 3E after only 10 min of incubation. After 2 h, CLSM images showed that the drug has completely left the mitochondria and diffused to the nucleus. Such rapid mitochondria trapping may result from the positive charge of P12. These results also manifested that most of the internalized DOX‐P18 could be selectively transported to mitochondria. As a result, MMPs in mitochondria turned DOX‐P18 into DOX‐P3. The observed trajectory analysis of DOX‐P18 was quite different from the pathway of a receptor‐mediated peptide‐based drug, that is, subject to lysosomal degradation. Colocalization of DOX and DAPI or lysosome/endosome/mitochondria tracker green (green) after 30 min co‐incubation with MDA‐MB‐231 cells showed that DOX was well‐distributed in the entire cell (Figure S13, Supporting Information).

The results of long‐time observation for the distribution of DOX‐P18 in Figure S14, Supporting Information indicated that DOX‐P3 could be retained in the nucleus, as evidenced by the high degree of colocalization between DAPI and DOX‐P18 after 12 h incubation in the obtained CLSM images. At 36 h, cells could not maintain a stable adhesion on the glass dish and would undergo a DOX‐P3‐induced apoptosis process. According to the above results, it could be concluded that DOX‐P18 conjugate could not only reduce DOX toxicity but would facilitate the entry of DOX units into the nucleus.

### 2.4 Antimigration and Invasion Assay

**Figure 4:**
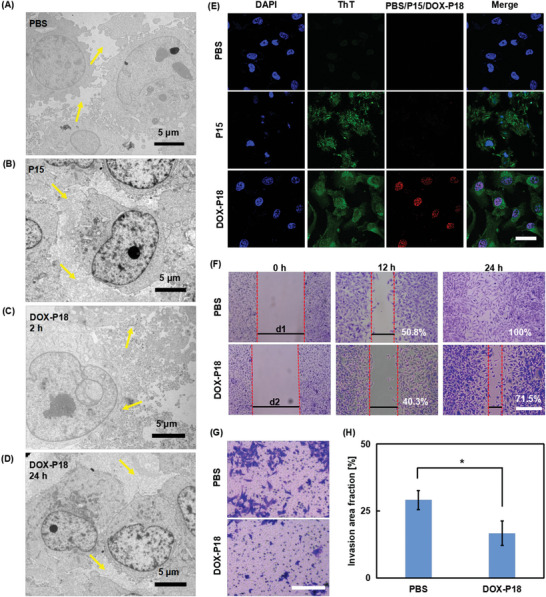
Anti‐metastasis evaluation of DOX‐P18. A–D) The ultrathin cell sections of PBS‐treated, P15‐treated, and DOX‐P18‐treated MDA‐MB‐231 cells. Yellow arrows indicate DOX‐P18 with different morphologies in the extracellular matrix. Scale bar: 5 µm. E) Extracellular and intracellular nanofibers detected by ThT fluorescence. Scale bar: 50 µm. F–H) Microscopy images of wound healing, invasion assay, and quantitative analysis of MDA‐MB‐231 cells. Scale bar: 100 µm. Data are presented as means ± SD (*n* = 3). * indicates *p* < 0.05.

To further study the inhibitory capability of DOX‐P18 for tumor invasion and metastasis, wound healing, and transwell invasion assays were performed. Before the antimigration and invasion assay evaluation, the morphological transformation of DOX‐P18 in MDA‐MB‐231 cells was investigated by TEM and confocal imaging. The extracellular and intracellular nanofibers self‐assembled from P15 were observed by cellular ultrathin sections of TEM (**Figure** **4**A–D). Moreover, thioflavin T (ThT) was used for nanofibers indicator imaging, due to its fluorescence resonance energy transfer effects. The fluorescence of ThT increases in P15‐treated cells further confirmed the assembly of P15 in MDA‐MB‐231 cells (Figure 4E).

The results of the wound healing assay for the antimigration study of DOX‐P18 on cell motility are shown in Figure 4F. A strong migration healing ability was exhibited on highly metastatic MDA‐MB‐231 cells of the PBS group 24 h after scratching, leaving nearly no scratch gaps to distinguish, which was defined as the 100% wound healing rate. Compared with 50.8% of the PBS group, the wound healing rate of DOX‐P18‐treatment group decreased to 40.3% at 12 h. The inhibition effect was further enhanced at 24 h and a coverage rate of 71.5% was observed which is much lower than that of the PBS group (100%). As shown in Figure 4G,H, the results of the transwell invasion assay indicated that MDA‐MD‐231 cells of the PBS group possess a high invasion ability to penetrate the Matrigel‐coated membrane. In sharp contrast, fewer MDA‐MD‐231 cells could invade the Matrigel in the DOX‐P18‐treated group, displaying a 50% inhibition on cell invasion. Taken together, the inhibited migration and invasion behaviors of MDA‐MB‐231 cells in the DOX‐P18 group than that of the PBS group suggested an antimetastatic potential through inhibiting migration and invasion of metastatic breast cancer cells.

### 2.5 Biocompatibility, Biodistribution, and Pharmacokinetics In Vivo

**Figure 5:**
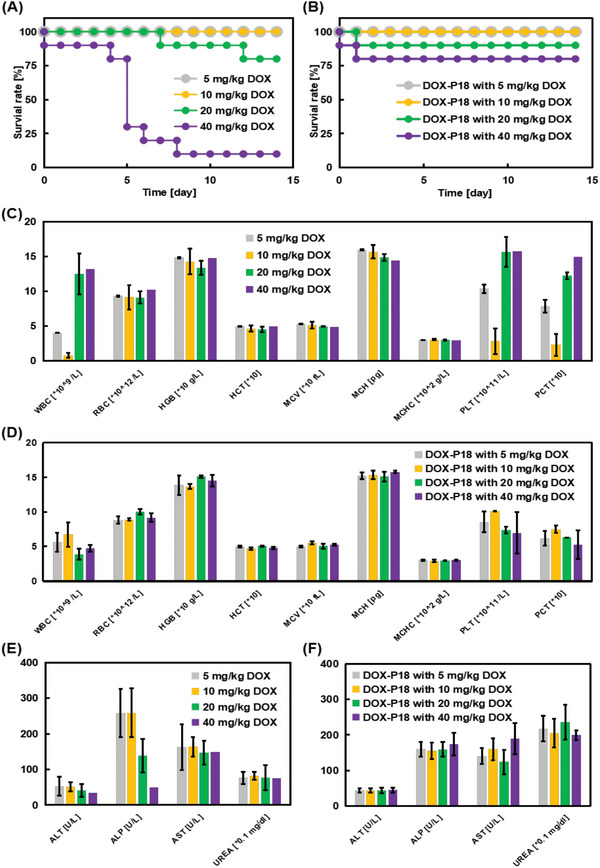
Biosafety study of DOX‐P18 in vivo. A,B) Survival rate of ICR mice during the maximum tolerated dose studies on DOX and DOX‐P18 (*n* = 10). C,D) Blood routine analysis of ICR mice injected with DOX and DOX‐P18 after 14 days. Data are presented as means ± SD (*n* = 3). E,F) Blood biochemical examination of ICR mice injected with DOX and DOX‐P18 after 14 days. Data are presented as means ± SD (*n* = 3).

To test the in vivo acute toxicity of transformable prodrug DOX‐P18, the maximum tolerated dose (MTD) in healthy ICR mice was determined. The survival rates of ICR mice injected with different concentrations of DOX and DOX‐P18 were recorded within 14 days in **Figure** **5**A,B. DOX showed persistent toxicity in vivo and would cause gradual mice death. However, the toxicity of DOX‐P18 was evidently milder that the massive acute mortality that occurred in the DOX‐treated group, even at a high DOX‐P18 dose of 200 mg kg^−1^ (equivalent to 40 mg kg^−1^ DOX). The single‐dose median lethal dose (LD_50_) of DOX was estimated to be 26.4 mg kg^−1^, while that of DOX‐P18 was 221.5 mg kg^−1^ (equivalent to 44.3 mg kg^−1^ DOX) (Table S4, Supporting Information), attaining a 67.8% improvement of biosafety.

The blood routine analysis results are shown in Figure 5C,D. At the dose of 10 mg kg^−1^ DOX, the indexes of white blood cell (WBC), platelet (PLT), and procalcitonin (PCT) in mice decreased remarkably, indicating some damage to the bone marrow hematopoietic cells in mice. Administrated with different doses of DOX‐P18, all blood routine indexes of the mice were not significantly different, exhibiting higher safety than free DOX. Data from blood biochemical analysis are shown in Figure 5E,F. With an increasing dose of DOX injected into ICR mice, the activity of alanine aminotransferase (ALT) and alkaline phosphatase (ALP) in mice gradually decreased, indicating that liver damage may have ensued. For a comprehensive comparison of the toxicity of DOX and DOX‐P18, histological analysis of major organs was performed 14 days after injection of single‐dose DOX and DOX‐P18 with different doses. As shown in Figure S15, Supporting Information, inflammatory cell infiltration progressively worsened in all groups as the injected dose increased. Specifically, disordered arrangement of myofilaments, glomerulosclerosis, and protein casts was noticed in the 40 mg kg^−1^ DOX group, while, there were no significant differences in major organs in DOX‐P18‐injected mice, even in the highest injected dose of 40 mg kg^−1^ DOX.

**Figure 6:**
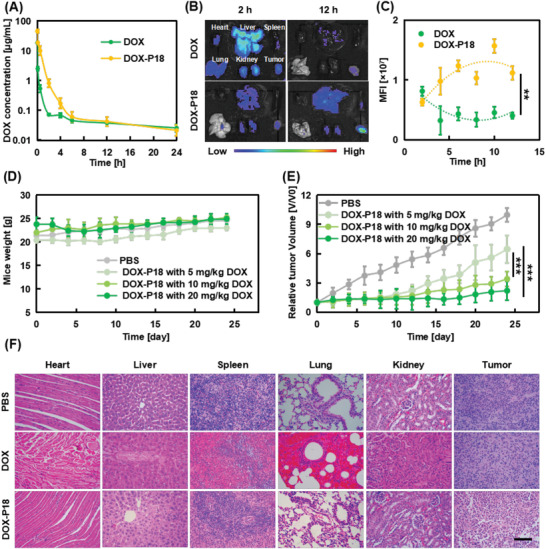
Biodistribution, pharmacokinetics and antitumor efficacy assays of DOX‐P18 on MDA‐MB‐231 tumor‐bearing nude mice. A) Plasma drug concentration in mice following tail‐vein injection of free DOX and DOX‐P18 (10 mg kg^−1^ DOX). Data are presented as means ± SD (*n* = 3). B) Ex vivo fluorescence images of major tissues and tumors with a single injection of DOX and DOX‐P18, 2 and 12 h later. C) The MFI of tumors at 2 and 12 h post‐injection. Data are presented as means ± SD (*n* = 3). D,E) The body weight and tumor volume of mice treated with DOX‐P18 of different doses. Data are presented as means ± SD (*n* = 5). F) Histological analysis of mice major organs after 24‐day treatment with DOX (5 mg kg^−1^) and DOX‐P18 (5 mg kg^−1^ DOX). Scale bar = 200 µm. ** indicates *p* < 0.01, and *** indicates *p* < 0.001.

To systematically understand the in vivo behaviors of transformable prodrug DOX‐P18, a pharmacokinetic study was carried out to quantitatively determine plasma drug concentration (**Figure** **6**A). Analyzed by Phoenix WinNonlin 8.3.5 software with PK Model, results revealed that the blood elimination half‐life time (t_1/2*β*_) values of DOX and DOX‐P18 were 14.9 and 10.5 h, respectively (Table S5, Supporting Information). The total plasma concentration (AUC) of the DOX‐P18 was four times higher than that of DOX and the plasma clearance (CL) of DOX‐P18 was only 1/4 of that of DOX. All the above results implied that prodrug DOX‐P18 could effectively improve the pharmacokinetics of DOX and prolong the circulation time in the blood, thereby improving the ability to effectively target tumor tissues.

To investigate the MMPs concentration in MDA‐MB‐231 tumor xenograft, an enzyme‐linked immunosorbent assay (ELISA) was conducted. The results showed that the measured concentrations of MMP‐2 and MMP‐9 were 9 and 7 ng mL^−1^, respectively, which was close to clinical data.^[^ ^19^ ^]^ Also, there have been reports suggesting that MMP‐9 level is a better marker than MMP‐2 in predicting breast cancer development and progression.^[^ ^20^ ^]^

To study the selective targeting of transformable prodrug DOX‐P18 on TNBC tumors, a dual xenograft model was developed with two different breast cancer cell lines MCF‐7 and MDA‐MB‐231. A much higher fluorescent signal of IR808 labeled NPY analogue P12 (IR808‐P12) in Figure S16, Supporting Information was observed on MDA‐MB‐231‐tumor bearing mice, which indicated a selectively targeting to TNBC tumors. To further evaluate the biodistribution of DOX and DOX‐P18 in tissues, MDA‐MB‐231 tumor‐bearing BALB/c nude mice were sacrificed after a single injection at different time points for ex vivo fluorescence imaging. As shown in Figure 6B,C, the highest DOX level was observed in the liver and moderate DOX uptake was found in the heart, lung, and kidney. With the prolongation of circulation time, the concentration of DOX in various organs gradually decreased, suggesting its rapid in vivo metabolism. On the other hand, DOX‐P18 attained the highest content in tissues at 6 h, indicating a longer duration in blood than DOX as a prodrug to avoid rapid clearance from circulation. Moreover, the fluorescence intensity in the tumor site increased after 6 h, which may suggest an effective in situ MMPs cleavage of DOX‐P18 to release DOX‐P3. To verify this observation, extracts from tumor tissues after 2 h intratumoral administration were examined by LC‐MS (Figure S17, Supporting Information). ESI (+) mass spectra (Figure S17C,D, Supporting Information) and ESI‐MS/MS spectra (Figure S17E,F, Supporting Information) confirmed that DOX‐P18 was substantially degraded at the G/V site in tumor tissues. Enhanced drug retention of DOX‐P18 was observed in tumors and the distribution of DOX‐P18 in other tissues reduced at 12 h after administration compared to DOX. The improved biodistribution indicated that DOX‐P18 possessed a strong tumor‐homing property, leading to less toxic side effects.

### 2.6 Antitumor and Anti‐Metastasis Efficiency In Vivo

The antitumor efficacy of transformable prodrug DOX‐P18 was evaluated in the MDA‐MB‐231 xenograft model. According to obtained LD_50_, high‐toxicity doses were excluded for the following in vivo anticancer evaluation. However, DOX at 10 and 20 mg kg^−1^ doses would also cause death due to the poor tolerance of tumor‐bearing nude mice (Figure S18, Supporting Information). To our delight, no death or obvious weight losses were observed in the DOX‐P18‐treated group even at a dosage of 100 mg kg^−1^ (equivalent to 20 mg kg^−1^ DOX) (Figure 6D). A modest effect of DOX‐P18 was achieved on tumor size reduction with an inhibition rate of ≈50% at a dose of 25 mg kg^−1^ (equivalent to 5 mg kg^−1^ DOX) (Figure 6E). With the increase of drug dose to 100 mg kg^−1^ (equivalent to 20 mg kg^−1^ DOX), DOX‐P18 showed significant inhibitory efficacy on MDA‐MB‐231 tumor growth. Figure 6F showed the histological analysis of major organs harvested 24 days after treatment with DOX (5 mg kg^−1^) and DOX‐P18 (5 mg kg^−1^ DOX). After five‐time administrations, cardiomyocytes were deformed in mice of DOX group, reflecting strong cardiotoxicity of DOX. Besides, spleen lymphocytes were reduced, and congested liver and lung tissues were observed. In the DOX‐P18 group, a pyknosis of the tumor cells appeared and the necrotic foci of the tumor tissues increased without obvious lesions in other organs. These results demonstrated the significant tumor suppression effect of DOX‐P18 with low side effects in the MDA‐MB‐231 tumor xenograft model.

**Figure 7:**
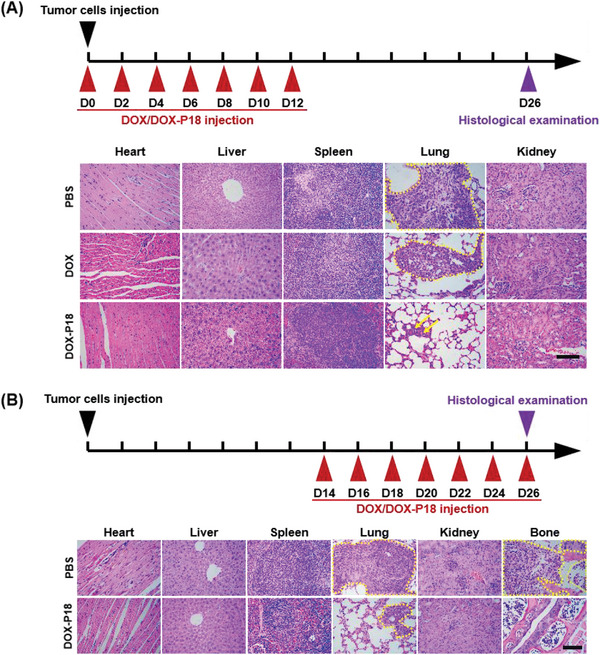
Evaluation of antitumor metastasis. A) Histological analysis of mice organs at D26 after seven administrations at D0, D2, D4, D6, D8, D10, and D12 of DOX (5 mg kg^−1^) and DOX‐P18 (5 mg kg^−1^ DOX). Scale bar = 200 µm. B) Histological analysis of mice major organs and bone tissues at D26 after seven administrations at D14, D16, D18, D20, D22, D24, and D26 of DOX‐P18 (5 mg kg^−1^ DOX). Yellow marks indicate tumor cells in tissues. Scale bar = 200 µm.

The anti‐metastatic efficiency of DOX‐P18 was evaluated in the MDA‐MB‐231 metastasis model. The results verified the successful establishment of the breast cancer metastasis model through in vivo and ex vivo fluorescence imaging and hematoxylin and eosin (H&E) staining analysis (Figures S19 and S20, Supporting Information). The metastatic foci could be extensively detected in the lungs at day 12 (D12) (Figures S19A and S20A, Supporting Information). Different degrees of bone metastasis occurred at day 26 (D26) (Figures S19B and S20B, Supporting Information). Metastasis prevention assessment was performed with a formulation (25 mg kg^−1^ DOX‐P18, equivalent to 5 mg kg^−1^ DOX) seven times at an interval of 2 days from day 0 (D0). To further elucidate the inhibition of tumor lung metastasis, tissues in each group were harvested for histological analysis on day 26. The obvious enhancement of the relative area (marked as yellow circle in **Figure** **7**A) of metastases in the PBS group depicted failures to control the progression of tumor metastasis. While limited number (marked as yellow arrows in Figure 7A) of cancer cells were found in the lungs of DOX‐P18‐treated mice, which proved the successful management of pulmonary tumor lesions.

At the same time, metastasis treatment assessment was performed with a formulation (25 mg kg^−1^ DOX‐P18, equivalent to 5 mg kg^−1^ DOX) seven times at an interval of two days from day 14 (D14). Compared to the severe infiltration of tumor cells in lung and bone tissues in the PBS group, DOX‐P18 exerted significant metastasis inhibition as indicated by the reduced intensity of cancer cells in lung and non‐pathological bone tissues in Figure 7B. However, no mice survived up to day 14 (D14) in the DOX‐treated group. Collectively, these observations demonstrated the chemotherapeutic superiority of DOX‐P18 on tumor metastasis management over free DOX.

## 3 Conclusion

We have developed a tumor microenvironment responsive transformable prodrug DOX‐P18, in which a novel NPY analogue was connected to chemotherapeutic DOX using MMPs‐cleavable peptide PLGVRG as a bridge. The prodrug showed reversible morphological transformation, desirable enzymatically triggered release, and incremental inhibitory activities on triple‐negative breast cancer cell line MDA‐MB‐231. Besides, the nest‐building behavior of the cleavage residue P15 generates an inhibition of tumor metastasis. In addition, DOX‐P18 exhibited higher biosafety with nearly two‐fold improved MTD compared to that of DOX on ICR mice. Pharmacokinetics data revealed a lower plasma clearance and higher bioavailability of DOX‐P18 in vivo. Improved biodistribution was verified on MDA‐MB‐231 tumor‐bearing nude mice where DOX‐P18 could efficiently enrich tumor tissues, leading to less toxic side effects. Notably, the significant antitumor and anti‐metastasis efficiency of DOX‐P18 with less toxicity was demonstrated in the MDA‐MB‐231 tumor xenograft and metastasis model. Further preclinical studies may warrant the potential application of prodrug DOX‐P18 as a specific targeted chemotherapeutic agent to achieve diversified biological functions for Y_1_R‐overexpressed triple‐negative breast cancer.

## 4 Experimental Section

### Acidic and Enzymatic Responsive of DOX‐P18

Morphology of DOX‐P18 in PBS 7.4, P15 in PBS 6.5, and DOX‐P18 treated with 0.1 µg mL^−1^ MMP‐9 were investigated by TEM, respectively. The size distribution of DOX‐P18 in PBS buffers with different pH (pH 7.4, 6.5, 6.0, and 5.0) was measured by DLS. The MMPs‐responsive cleavage was conducted in 1 mg mL^−1^ DOX‐P18 with 1 µg mL^−1^ MMP‐2 or MMP‐9 at 37 °C. The enzymatic reaction process was monitored by HPLC at 6, 12, 24, and 72 h. The results of MMPs‐responsive cleavage of DOX‐P18 were confirmed by Q‐TOF‐MS.

A fluorescence spectrophotometer was used to monitor the process of the enzymatic reaction of MMPs to DOX‐P18. After incubation of DOX‐P18 with MMPs for 12 and 24 h, fluorescence intensities at 592 nm of the buffer mixture (containing 0.1 µg mL^−1^ MMP‐2 or MMP‐9, 20 µg mL^−1^ DOX‐P18) were also investigated to quantify the cleavage efficiency (*Ex/Em* = 488/510–800 nm).

### Flow Cytometry Test

Flow cytometry test was performed to detect the cellular uptake of DOX‐P18 and cellular MMPs‐responsive drug release. MDA‐MB‐231 cells were plated in 6‐well plates (2 × 10^5^ cells per well) for 24 h. To study the cellular uptake mechanism, DOX‐P18 was diluted in fresh DMEM with 10% FBS to the final concentration of 40 µg mL^−1^ (calculated according to DOX content) and separately co‐incubated with MDA‐MB‐231 cells under 4 °C, lysosomotropic agent chloroquine (CQ, 100 µM), Y_1_R antagonist (BIBP3304, 20 µM), chlorpromazine (CPZ, 30 µM), amiloride (50 µM), and methyl‐*β*‐cyclodextrin (M*β*CD, 5 mM) treatment for 1 h at 37 °C. After centrifugation and washing, DOX‐P18‐treated cells were collected in 5 mL polypropylene round‐bottom tubes for flow cytometry test. The mean fluorescence intensity (MFI) of 1 × 10^4^ cells was detected by flow cytometry (FACSCalibur, BD, USA). Besides, 5 mM ethylenediaminetetraacetic acid (EDTA) purchased from Sigma was used as a model inhibitor to inhibit MMPs activity.

### CLSM Observation

1 × 10^5^ MDA‐MB‐231 cells were seeded in confocal dishes for 24 h. 5 µg mL^−1^ DOX‐P18 was added to incubate with MDA‐MB‐231 cells at 37 °C for 12, 24, and 48 h. After fixing with 4% PFA for 30 min in the dark, the cells were stained with DAPI (C1002, Beyotime, China) at 37 °C for another 30 min. Eventually, the cells were imaged by CLSM in dual fluorescence mode, blue channel for DAPI (*Ex/Em*: 408/420–480 nm) and red channel for DOX‐P18 (*Ex/Em*: 488/550–600 nm). For localization of DOX‐P18 in subcellular organelles, lysosome, endosome, and mitochondria were separately stained with early endosome marker (ab109009), lyso‐tracker green (C1047S, Beyotime, China), and mito‐tracker green (C1048, Beyotime, China) for 30 min.

### ThT Assay

1 × 10^5^ cells were cultured overnight in confocal dishes. PBS, P15, or DOX‐P18 (5 µg mL^−1^, 1 mL) was added and cultured for 24 h. Then, the cells were washed three times. ThT solution (4 µM) was used to detect the nanofibers with an emission spectrum shift from 440 to 490 nm.

### Cell Viability Studies

Cytotoxicity of DOX, DOX‐P3, and DOX‐P18 was tested by cell counting kit‐8 (CCK‐8) assay. MDA‐MB‐231 cells were incubated in 96‐well plates for 24 h (1 × 10^4^ cells per well). DOX and P18 of different concentrations (0.3125, 0.625, 1.25, 2.5, 5, 10, 20, and 40 µg mL^−1^) were co‐incubated with the MDA‐MB‐231 cells for 24 h at 37 °C. Cytotoxicity of DOX‐P3 and DOX‐P18 (0.3125, 0.625, 1.25, 2.5, 5, 10, 20, and 40 µg mL^−1^) was studied at different periods of time (24, 36, and 48 h). At the appointed time, 10 µL CCK‐8 solution was dropped into each well and further co‐incubated with treated cells for 2 h. The absorbance values at 450 nm were measured by an automated plate reader (iMark (168‐1130), Biorad, USA). The IC_50_ values were obtained from Microsoft Excel's FORECAST formula.

### Wound Healing Experiment

Wound healing culture inserts were tightly stuck on 12‐well plates and 2 × 10^4^ MDA‐MB‐231 cells were seeded in the gap of the culture inserts to incubate for 24 h. Once the cells covered 80% of the dish surface, the culture inserts were removed and fresh culture medium with 5 µg mL^−1^ DOX‐P18 was added. After incubation for 12 and 24 h, the wound healing area was photographed by a microscope (DFC 450C, Leica, Germany).

### Transwell Experiment

1 × 10^6^ MDA‐MB‐231 cells were suspended on the top chambers of the transwell with Matrigel. Cells were cultured in DMEM without FBS overnight. 500 µL DMEM with 10% FBS and 1 µg mL^−1^ DOX‐P18 was added to the lower chambers. After incubation for 24 h, the cells on the upper wells were cautiously erased with a cotton swab. Cells on the lower surface were fixed with 4% PFA and stained with crystal violet. After multiple washing with PBS, the cells that passed through the membrane were recorded with a microscope (DFC 450C, Leica, Germany).

### Tumor Models

All the animal experimental procedures were recognized and approved by the Regional Ethics Committee for Animal Experiments at Ningbo University, China (Permit NO. SYXK (Zhe) 2019‐0005). The triple‐negative breast cancer (TNBC) xenograft tumors were established on female BALB/c nude mice (≈15 g) by subcutaneous injection of MDA‐MB‐231 cells (5 × 10^6^ cells in each mouse). TNBC metastasis models were constructed by injecting MDA‐MB‐231‐luc cells (1 × 10^5^ cells in each mouse) through the tail vein to female BALB/c nude mice. The day that MDA‐MB‐231‐luc was injected was set as D0. In vivo imaging system (IVIS) (Lumina XRMS Ш, PerkinElmer, USA) was utilized to identify the tumor metastasis at D12.

### Maximum Tolerated Dose Studies

To evaluate the toxicity in vivo, the maximum tolerated dose (MTD) of DOX‐P18 on healthy ICR mice (≈20 g weight) was determined. The single‐dose MDT of DOX intravenously injected was set at 5, 10, 20, and 40 mg kg^−1^ (5 mg kg^−1^ equivalent to 1 mg mL^−1^) referring to previous findings, while the studied single‐dose MDT of DOX‐P18 was 50, 100, 200, and 400 mg kg^−1^ (50 mg/kg equivalent to 10 mg mL^−1^). Animal survival of each group (*n* = 10, 5 female and 5 male) was continuously recorded for 14 days. Eventually, the evaluation and calculation of the median lethal dose (LD_50_) were completed with these collected data using the Bliss rule. The 14‐day survival curve was also plotted to clarify the acute toxicity of DOX and DOX‐P18.

### Pharmacokinetics

Four‐week‐old ICR female mice were injected with a single dose of 10 mg kg^−1^ DOX and DOX‐P18 (calculated according to DOX content). At 1, 5, and 30 min and 2, 4, 6, 12, and 24 h post‐injection, the blood was centrifuged to collect plasma and the drug was extracted using methanol solution (methanol: H_2_O = 4:1 for DOX) and ACN solution (ACN: H_2_O = 3:1 for DOX‐P18). DOX and DOX‐P18 standards were added to the freshly obtained plasma to determine the extraction recovery. The drug contents were quantified by HPLC (1260 Infinity II, Agilent, USA) as described in the methods of characterization in Supporting Information and the pharmacokinetic parameters were analyzed using the Phoenix WinNonlin 8.3.5 software.

### Biodistribution Studies

To investigate the biodistribution and tumor targeting ability in vivo, DOX and DOX‐P18 were injected intravenously into MDA‐MB‐231 tumor‐bearing nude mice. At 2, 4, 6, 8, 10, and 12 h post‐injection, the major organs and tumor were taken out and fluorescence signals were captured by IVIS.

### In Situ MMPs‐Responsive Cleavage of DOX‐P18

MMPs concentration in tumor tissues was tested by enzyme‐linked immunosorbent assay (ELISA). Human MMP‐2 (SEKH‐0253) and MMP‐9 (SEKH‐0257) ELISA kits were purchased from Solarbio Life Sciences Ltd. Co. (Beijing, China). Briefly, tumor tissues were mechanically crushed and then lysed with the RIPA lysate solution containing protease inhibitors. The supernatants were used for detecting MMP‐2 and MMP‐9.

For in situ MMPs‐responsive cleavage of DOX‐P18, 1 mg mL^−1^ DOX‐P18 (50 µL) was injected into the tumor of BALB/c nude mice. The tumor was peeled off 2 h later and an automatic homogenizer (JXFSTPRP‐32L, Jingxin, China) was applied to grind and crush the tumor tissues. Proteins in tissue were precipitated with ACN solution (ACN: H_2_O = 3:1) and the supernatant was collected for UPLC/Q‐TOF‐MS analysis. Analytical UPLC system (Nexera X2 LC‐30, Shimadzu, Japan) equipped with an AdvanceBio Peptide Map column (2.7 µm, 2.1 × 150 mm, 0.25 mL min^−1^; Agilent, USA) was used by applying a linear gradient of 30% to 52% (v/v) eluent B in eluent A in 14 min, 52% to 90% (v/v) eluent B in eluent A in 6 min. 5 µL sample was injected and detection was performed at 500 nm. For Q‐TOF‐MS (Triple TOF 4600, AB Sciex, USA), ESI (+) mode was applied. Data ranging from 500 to 502 nm were collected and processed with Analyst TF 1.7 software.

### In Vivo Antitumor Evaluation

When the tumor volume of BALB/c mice reached ≈70 mm^3^ (V = L × W × W/2), the tumor‐bearing mice were randomly divided into seven groups (*n* = 5 for each group). A series of dosages (5, 10, and 20 mg kg^−1^, calculated according to DOX content) of DOX and DOX‐P18 were used for the in vivo study. 100 µL drug was injected intravenously through the tail vein every other day for a total of five times. The day that the first injection was given was set as D0. Tumor size and mouse weight were measured every 2 days from the day that treatment started until D26.

For the TNBC metastasis model, two kinds of treatments were developed. To inhibit TNBC metastasis, in the first method, DOX‐P18 (5 mg kg^−1^, 100 µL) was injected at D0, D2, D4, D6, D8, D10, and D12. After seven times of injections, continued observation of the mice was carried out. In the other method, DOX‐P18 (5 mg kg^−1^, 100 µL) was administered at D14, D16, D18, D20, D22, D24, and D26. In vivo imaging system (IVIS Lumina XRMS Ш, PerkinElmer, USA) was utilized again to estimate the tumor metastasis at D26. Meanwhile, the mice were sacrificed and the main organs (heart, livers, spleen, lung, and kidney) were separated for histological analysis.

### Statistical Analysis

All data were collected in triplicate and reported as means ± SD. Comparison between the groups was performed using a one‐tailed *t*‐test. One‐way analysis of variance was used, respectively, with *p < 0.05, **p < 0.01, and ***p < 0.001.

## Conflict of Interest

The authors declare no conflict of interest.

## Supporting information

Supporting Information

## Data Availability

The data that support the findings of this study are available from the corresponding author upon reasonable request.
