# Supplementary material for: Transformable Neuropeptide Prodrug with Tumor Microenvironment Responsiveness for Tumor Growth and Metastasis Inhibition of Triple‐Negative Breast Cancer
Source: Adv Sci (Weinh). 2023 May 5;10(21):2300545. doi: 10.1002/advs.202300545 (PMC10375191; doi:10.1002/advs.202300545)
Supplement: Supplementary file 1 — Supporting Information [file ADVS-10-2300545-s001.pdf]

ADVANCED SCIENCE Open Access

Supporting Information

for *Adv. Sci.*, DOI 10.1002/advs.202300545

Transformable Neuropeptide Prodrug with Tumor Microenvironment Responsiveness for Tumor Growth and Metastasis Inhibition of Triple-Negative Breast Cancer

Yi Cao, Xiaojiao Ge, Xueli Zhu, Yingying Han, Pin Wang, Ozioma Udochukwu Akakuru, Aiguo Wu and Juan Li**

Supporting Information

Transformable Neuropeptide Prodrug with Tumor Microenvironment Responsiveness for Tumor Growth and Metastasis Inhibition of Triple-Negative Breast Cancer

Yi Cao, [a, b] *Xiaojiao Ge,* [a] *Xueli Zhu,* [a] *Yingying Han,* [a] *Pin Wang,* [a, b] Ozioma Udochukwu Akakuru, [a] *Aiguo Wu,** [a] *Juan Li,** [a]

[a] Cixi Institute of Biomedical Engineering, International Cooperation Base of Biomedical Materials Technology and Application, CAS Key Laboratory of Magnetic Materials and Devices, Zhejiang Engineering Research Center for Biomedical Materials, Ningbo Institute of Materials Technology and Engineering, Chinese Academy of Sciences, Ningbo 315201, P. R. China

[b] University of Chinese Academy of Sciences, Beijing 100049, P.R. China

Email: aiguo@nimte.ac.cn

lij@nimte.ac.cn

Table of Contents

Table of Contents......2
Materials......3
Reagents and Cell Lines......3
Methods......4
Synthesis and Characterization......4
Peptide Synthesis......4
DOX-P3 and DOX-P18 Synthesis......4
Purification......5
Characterization......5
Immunocytochemistry......6
Western Blot Analysis......7
Immunohistochemistry and Histological Analysis......7
Supporting Figures......9
Scheme S1......9
Figure S1......10
Figure S2......11
Figure S3......12
Figure S4......13
Figure S5......14
Figure S6......15
Figure S7......16
Figure S8......17
Figure S9......18
Figure S10......19
Figure S11. 20
Figure S12......21
Figure S13......22
Figure S14. 23
Figure S15......24
Figure S16......25
Figure S17......26
Figure S18......27
Figure S19......28
Figure S20. 29
Table S1......30
Table S2......31
Table S3......32
Table S4......33
Table S5......34

Materials

Reagents and Cell Lines

Doxorubicin hydrochloride (DOX·HCl), trifluoroacetic acid (TFA), and anhydrous N,N-Dimethylformamide (DMF) were purchased from Aladdin (Shanghai, China). Thioanisole (TA), 1,2-ethanedithiol (EDT), and phenol were obtained from Macklin (Shanghai, China). 4-methylmorpholine (NMM), N,N'-diisopropylcarbodiimide (DIC), and 1-hydroxybenzotriazole (HOBt) were purchased from OKA (Beijing, China). Rink amide methylbenzhydrylamine (MBHA) resin, Wang resin and all Fmoc-protected amino acids were obtained from NJPeptide (Nanjing, China). Ethanol, diethyl ether, acetonitrile (ACN), and methanol were purchased from Sinopharm Chemical Reagent Co., Ltd (Shanghai, China). Piperidine, p-nitrophenol, and succinic anhydride (Succ) were obtained from Sigma-Aldrich (Shanghai, China). Recombinant human matrix metalloproteinase-2 (MMP-2) and matrix metalloproteinase-9 (MMP-9) produced by Novoprotein Scientific Inc. (Shanghai, China) are supplied as a 0.2 μm filtered buffer mixture of 20 mM Tris-HCl, 2 mM $CaCl_2$, 150 mM NaCl, 0.05 % Brij 35 (w/v), pH 7.5, and their specific activity is >1300 pmol/min/μg as measured under the described conditions. All commercial reagents and HPLC solvents were used with no further purification.

Human breast cancer cell line MCF-7, MDA-MB-231, MDA-MB-231-luc, and human mammary epithelial cell line MCF-10A were purchased from the American Type Culture Collection (ATCC). MCF-7, MDA-MB-231, and MDA-MB-231-luc cells were cultured in standard Dulbecco's modified Eagle's medium (DMEM)

supplied with 10 % (*v/v*) FBS. MCF-10A cells were cultured in DMEM/F-12 with 5 % horse serum, 10 μg/mL insulin, 0.5 μg/mL hydrocortisone, 20 ng/mL epidermal growth factor, and 100 ng/mL cholera toxin. All cell lines were cultured under a humidified atmosphere at 37 °C and 5 % CO_2.

Methods

Synthesis and Characterization

Peptide-DOX conjugates were synthesized using succinic acid (Succ) as a linker. Peptide and DOX were attached to Succ respectively through the N-terminal of proline and free amino group of DOX by automated solid phase peptide synthesis (SPPS).

Peptide Synthesis. Tripeptides P3 (Sequence: NH_2-PLG-COOH) and C-terminally amidated peptides P18 (Sequence: NH_2-PLGVRGRHYNN PIWRQRY-$CONH_2$) were synthesized on a microwave synthesizer (Biotage Initiator$^+$, Biotage, Sweden). Reactive side chains of amino acids were protected by *tert*-butyl (^{t}Bu for Tyr), trityl (Trt for Asn, Gln, His), 2,2,4,6,7-pentamethyldihydrobenzofuran-5-sulfonyl (Pbf for Arg), and *tert*-butoxycarbonyl (Boc for Trp). For automated synthesis, Fmoc deprotection was performed twice with 25 % (v/v) piperidine in DMF for 3 min (Temperature: RT, Vortex Rate: 1200 RPM). Coupling reactions were carried out three times with 3 equimolar Fmoc-protected amino acid, in situ activated with equimolar amounts of HOBt and DIC in DMF for 5 min (Temperature: 75 °C, Vortex Rate: 1200 RPM).

DOX-P3 and DOX-P18 Synthesis. Peptides P3 or P18 on resin were connected with

Succ after Fmoc deprotection and carboxyl group of Succ was actived by DIC to get p-nitrophenol conjugated. Peptides P3 and P18 connected with Succ and p-nitrophenol were cleaved from resin by using TFA/H_2O/TA/phenol/EDT (82.5:5:5:5:2.5, v/v/v/v/v) for 2 h (Temperature: room temperature). Furtherly, crude peptide derivatives were precipitated from ice-cold diethyl ether, washed and collected by centrifugation and lyophilization. DOX was finnaly coupled with the peptide derivatives obtained in the previous step by using standard DIC/HOBt activation.

Purification. Crude product purification was performed by preparative RP-HPLC on a Hanbon RP-HPLC system (NS4000, Huaian, China) with an EP-C18 column (10 μm, 250 × 21.2 mm, 10 mL/min; Galaksil, China) and a linear gradient system containing 0.1 % (v/v) TFA in water (eluent A) and ACN (eluent B).

Characterization. DOX-P3 and DOX-P18 were characterized by analytical high performance liquid chromatograph (HPLC) (1260 Infinity II, Agilent, USA) and ultra-high performance liquid chromatography quadrupole time-of-flight mass spectrometer (UPLC/Q-TOF-MS) (Triple TOF 4600, AB Sciex, USA). For analytical HPLC, 1260 Infinity II system equipped with a InfinityLab Poroshell 120 EC-C18 (4 μm, 4.6 × 100 mm, 1 mL/min; Agilent, USA) column was used by applying a linear gradient of 5 % to 95 % (v/v) eluent B in eluent A in 20 min. 5-μL DOX-P3 or DOX-P18 solution was injected and detection was performed at 500 nm. For data analysis, peaks were integrated by using Agilent OpenLab CDS ChemStation. For UPLC/Q-TOF-MS, positive electrospray ionization (ESI^+) mode was applied. Datas

were collected ranging from 500 to 1000 m/z and processed with Analyst TF 1.7 software.

The optical properties of DOX, DOX-P3, and DOX-P18 were studied by a UV-vis spectrophotometer (Lambda 950, PerkinElmer, USA) and a fluorescence spectrophotometer (FL3-111, Hitachi, Japan). UV-vis spectra of DOX, DOX-P3, and DOX-P18 (Concentration: 1, 2, 2.5, 5, 10, 20, 25, 50, and 100 μg/mL) were recorded ranging from 300 to 700 nm. Fluorescence spectra of DOX, DOX-P3, and DOX-P18 (Concentration: 2.5 μg/mL) were determined from 500 to 800 nm at an excitation (Ex) wavelength of 488 nm.

Immunocytochemistry

Immunocytochemistry analysis was performed to evaluate the MMPs and Y_1R expression on MCF-7, MDA-MB-231, and MCF-10A cell lines. 1×10^5 cells were plated in confocal dishes and incubated for 24 h, then rinsed with PBS for three times. After being fixed with 4 % paraformaldehyde (PFA) for 30 min at room temperature, these cells were permeabilized with 0.3 % Triton X-100 (T9284, Sigma, USA) in PBS for 20 min and blocked with 1 % BSA (A104912, Aladdin, China) for 1 h at 37 ℃. Afterwards, blocked cells were incubated with rabbit polyclonal anti-MMP-2 antibody (1:200; A6247, ABclonal, China), recombinant anti-MMP9 antibody (1:100; ab228402, Abcam, England), and anti-Y_1R antibody (1:200; ab219266, Abcam, England) for 1 h at 37 ℃. Subsequently, PBS was applied to remove the uncombined antibody and goat anti-rabbit IgG H&L (Alexa Fluor® 488) secondary antibody (1:2000; ab150077, Abcam, England) was added to incubated with cells for 30 min at

37 °C. The nuclei of the cells was stained with DAPI (C1005, Beyotime, China). Images were captured using a confocal laser scanning microscopy (CLSM) (TCS SP5 II, Leica, Germany).

Western Blot Analysis

MCF-7, MDA-MB-231, and MCF-10A cells were separately treated with RIPA lysis buffer (P0013, Beyotime, China) containing protease inhibitors and PMSF to get total cellular proteins extracted. Later, BCA protein assay kit (P0012, Beyotime, China) was used to quantify the total cellular protein. Quantified protein (~ 40 μg) was electrophoresed on a 10 % (w/v) SDS-PAGE gel at 120 V and then transferred to polyvinylidene fluoride (PVDF) membranes. Rabbit polyclonal anti-MMP-2 antibody (1:1000; A6247, ABclonal, China), anti-MMP-9 antibody (1:1000; ab228402, Abcam, England), and anti-Y_1R antibody (1:1000; ab91262, Abcam, England) were incubated with the protein retained PVDF membranes at 4 °C overnight. Rabbit polyclonal anti-GAPDH antibody (1:4000; D110016, Sangon Biotech, China) was selected as an internal reference. Protein bands were visualized by chemiluminescence on an automatic fluorescence/chemiluminescence image analysis system (5200, Tanon, China).

Immunohistochemistry and Histological Analysis

Immunohistochemistry (IHC) staining was performed to identify the expression of MMP-2, MMP-9, and Y_1R on MCF-7 and MDA-MB-231 subcutaneous xenotransplanted tumors. Fresh tumor tissues were harvested and immobilized in 4 % PFA for a week. Then, tissues went through 100 %, 95 %, and 80 % ethanol orderly to

get dehydrated. Later, paraffin-embedded samples were cut into 5 μm sections. The anti-MMP-2 (1:100; A6247, ABclonal, China), anti-MMP-9 antibody (1:2000; ab228402, Abcam, England), and anti-Y_1R antibody (1:200; ab219266, Abcam, England) were used in IHC staining. Universal secondary antibody kit used was from Zsbio (PV-6000, Zsbio, China). Hematoxylin and eosin (H&E) staining was conducted for histological observation. All images were recorded by fluorescence microscopy (DFC 450C, Leica, Germany).

Supporting Figures

Scheme S1. Synthesis Routes of DOX-P18 and DOX-P3

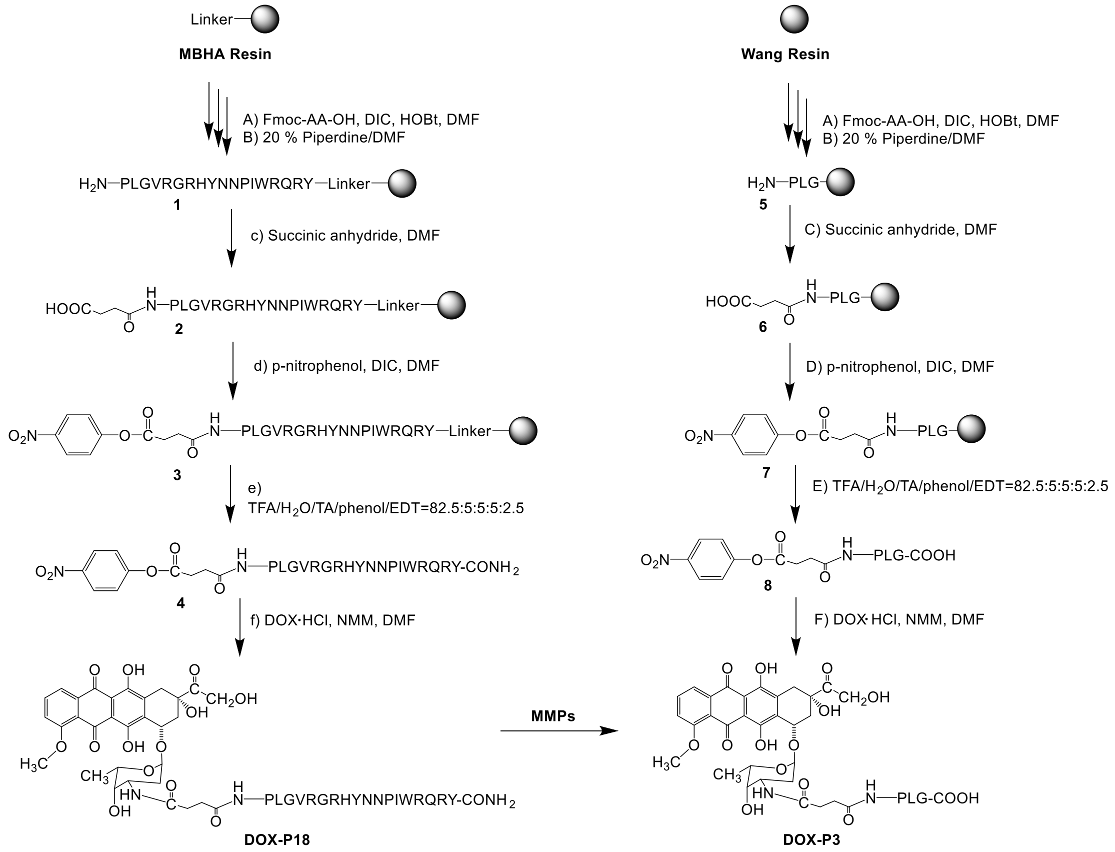

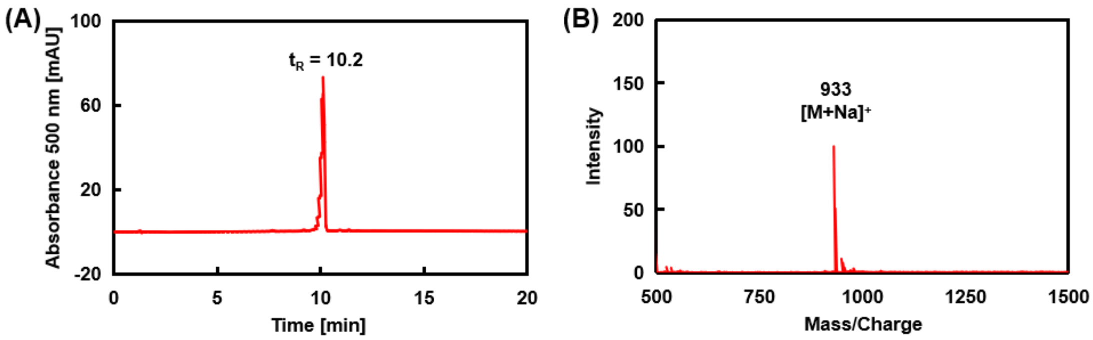

Figure S1. (A) Analytical HPLC of DOX-P3 using a InfinityLab Poroshell 120 EC-C18 column with a linear gradient of 5 % to 95 % (*v/v*) eluent B in eluent A in 20 min. (B) ESI (+) mass spectra of DOX-P3 recorded in the presence of Na^+.

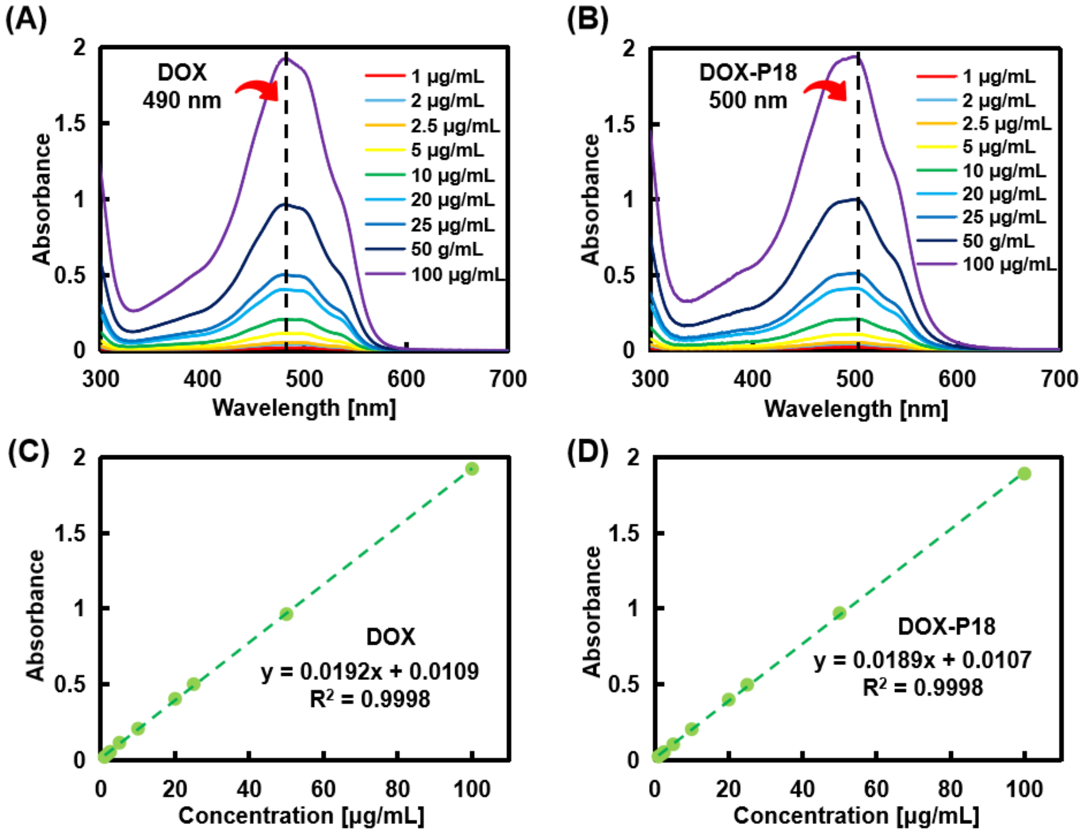

Figure S2. (A and B) UV-vis spectra of DOX and DOX-P18. (C and D) Concentration-absorbance standard curves of DOX and DOX-P18 from 1 to 100 μg/mL.

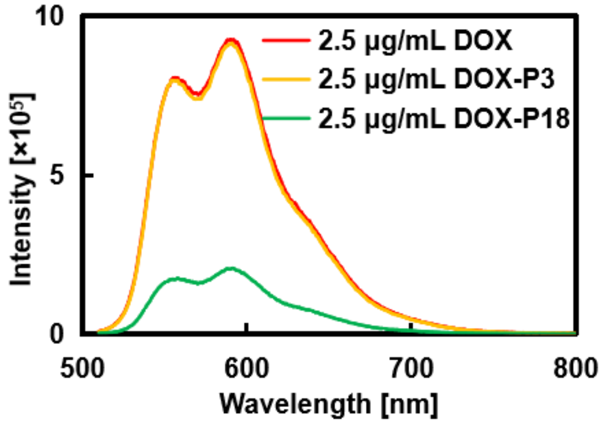

Figure S3. Fluorescence spectra of DOX, DOX-P3, and DOX-P18 at 2.5 μg/mL (Ex/Em = 488 nm/510-800 nm).

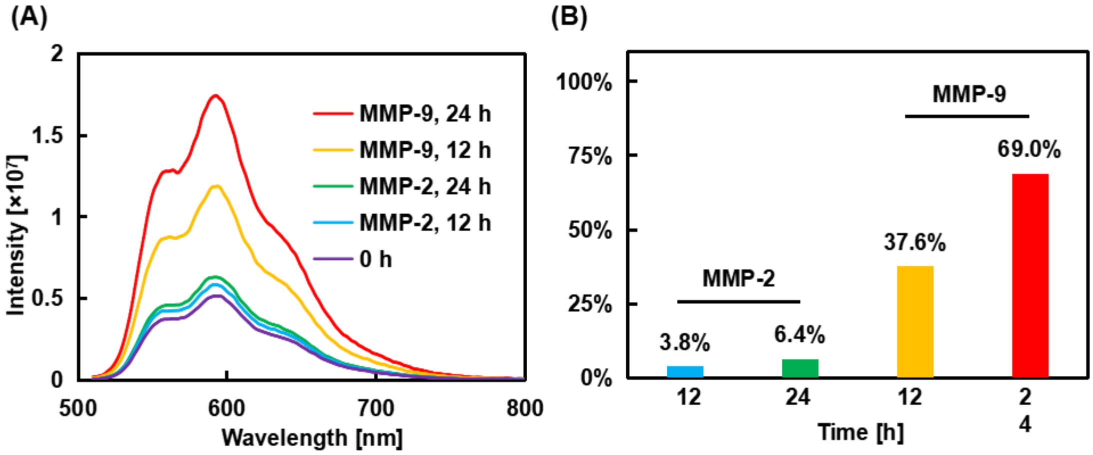

Figure S4. (A) Fluorescence spectra of DOX-P18 (20 μg/mL) after 12 and 24 h incubation with 0.1 μg/mL MMP-2 or MMP-9 (Ex/Em = 488 nm/510-800 nm). (B) Cleavage efficiency of MMP-2 and MMP-9 quantified by fluorescence intensities at 592 nm.

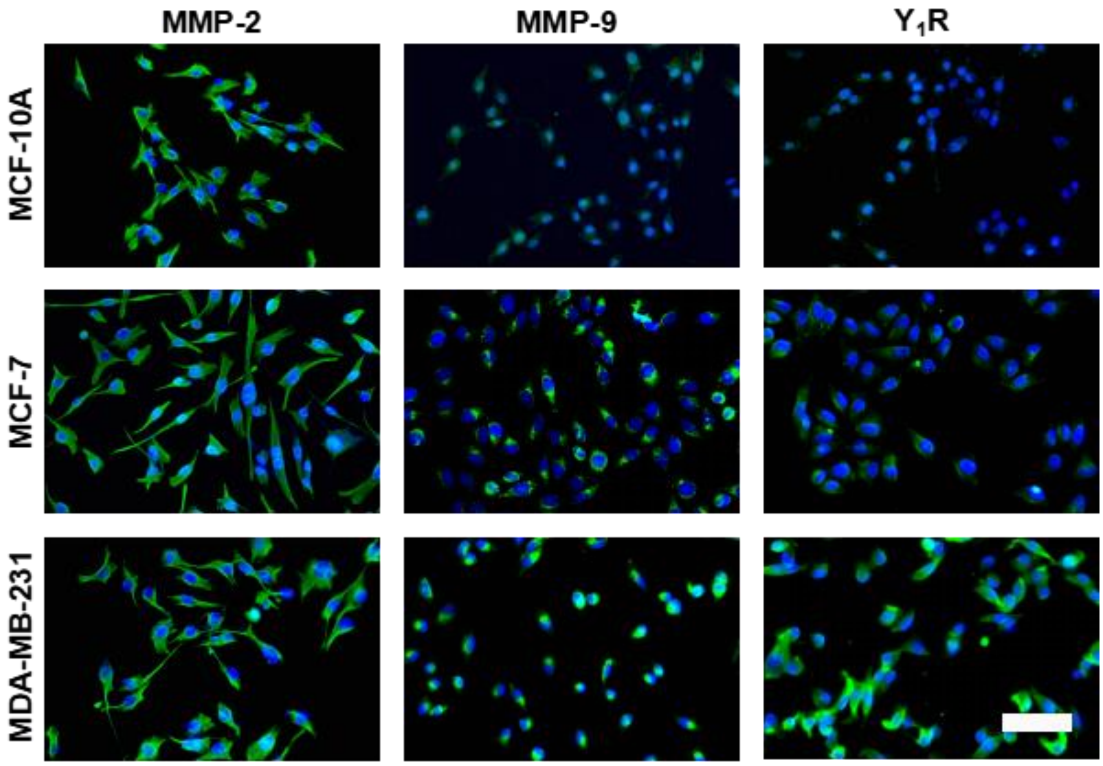

Figure S5. Immunocytochemistry assay of MMP-2, MMP-9, and Y_1R in MCF-10A, MCF-7, and MDA-MB-231 cell lines. Scale bar: 100 μm.

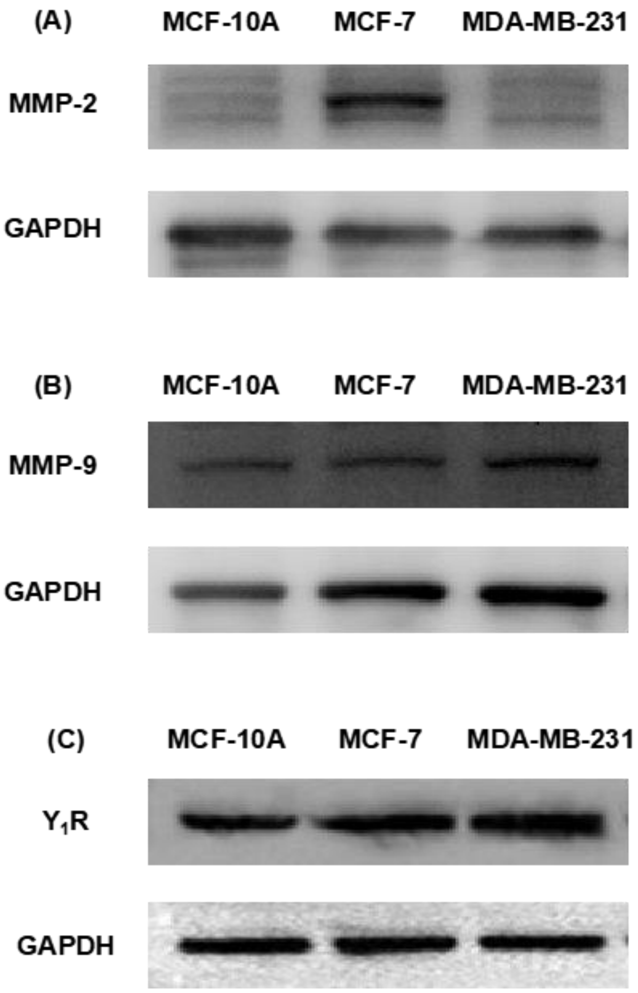

Figure S6. (A, B, C) Western blot analysis of MMP-2, MMP-9, and Y_1R in MCF-10A, MCF-7, and MDA-MB-231 cell lines.

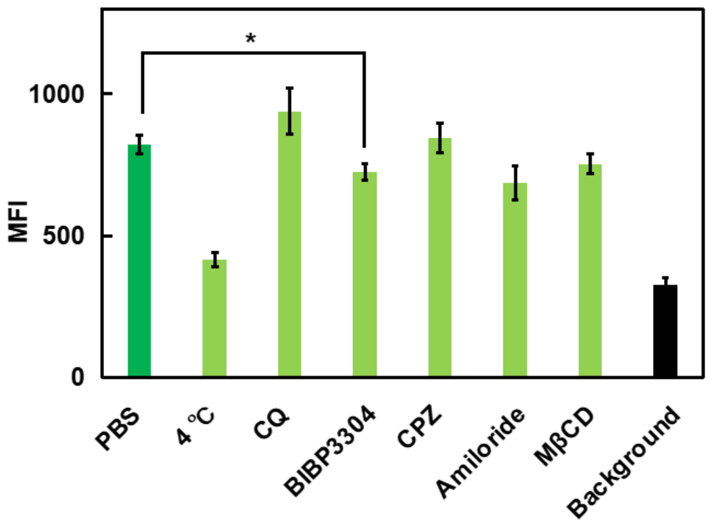

Figure S7. Flow cytometry assay of the cellular uptake of DOX-P18 under 4 °C, chloroquine (CQ), Y_1R antagonist (BIBP3304), chlorpromazine (CPZ), amiloride, and methyl-β-cyclodextrin (MβCD) treatment. The control is the mean fluorescence intensity (MFI) of MDA-MB-231 cells treated with PBS at 37 °C for 1 h. Data are presented as means ± standard deviations (n = 3). * indicates $p < 0.05$.

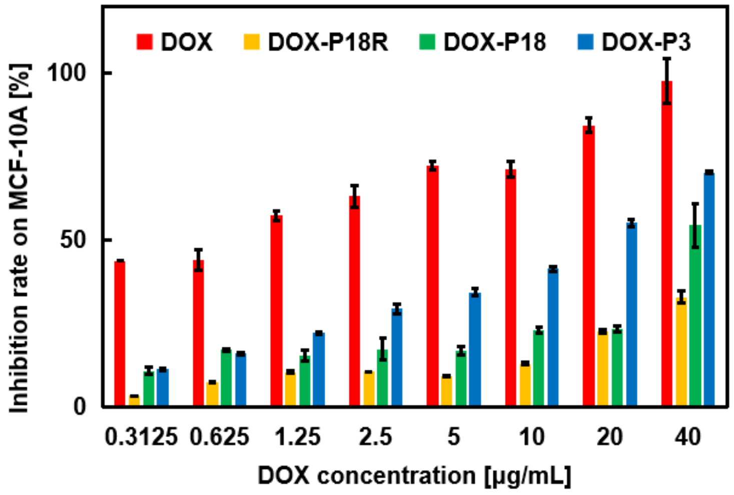

Figure S8. Cytotoxicity of DOX, DOX-P18R, DOX-P18, and DOX-P3 on human mammary epithelial cell line MCF-10A after 24 h incubation. Data are presented as means ± standard deviations (n = 3).

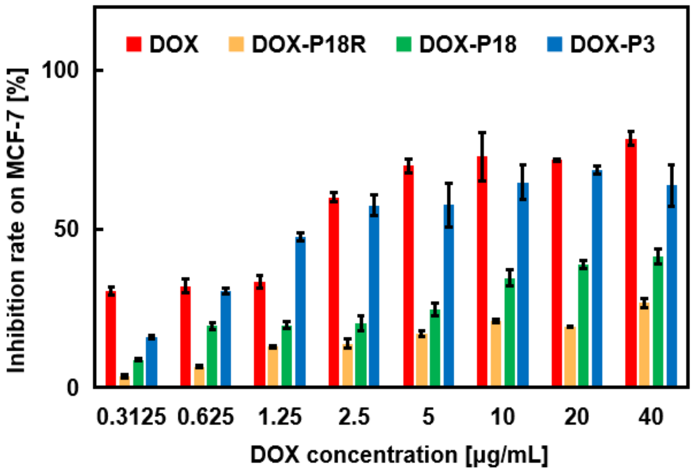

Figure S9. Cytotoxicity of DOX, DOX-P18R, DOX-P18, and DOX-P3 on human breast cancer cell line MCF-7 after 24 h incubation. Data are presented as means ± standard deviations (n = 3).

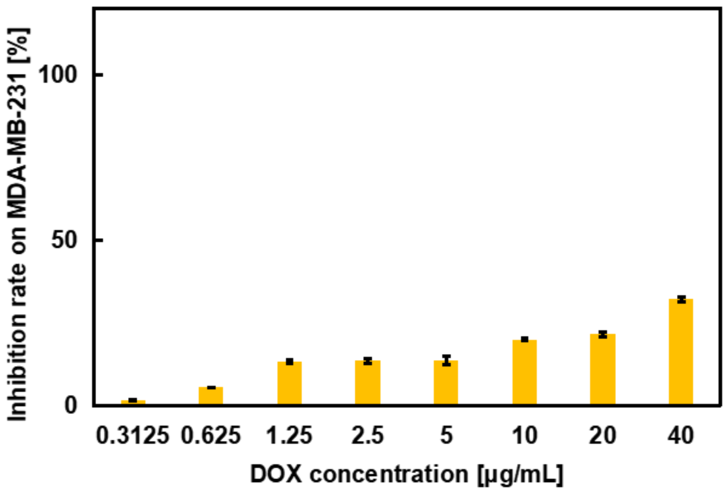

Figure S10. Cytotoxicity of DOX-P18R on human triple-negative breast cancer cell line MDA-MB-231 after 24 h incubation. Data are presented as means ± standard deviations (n = 3).

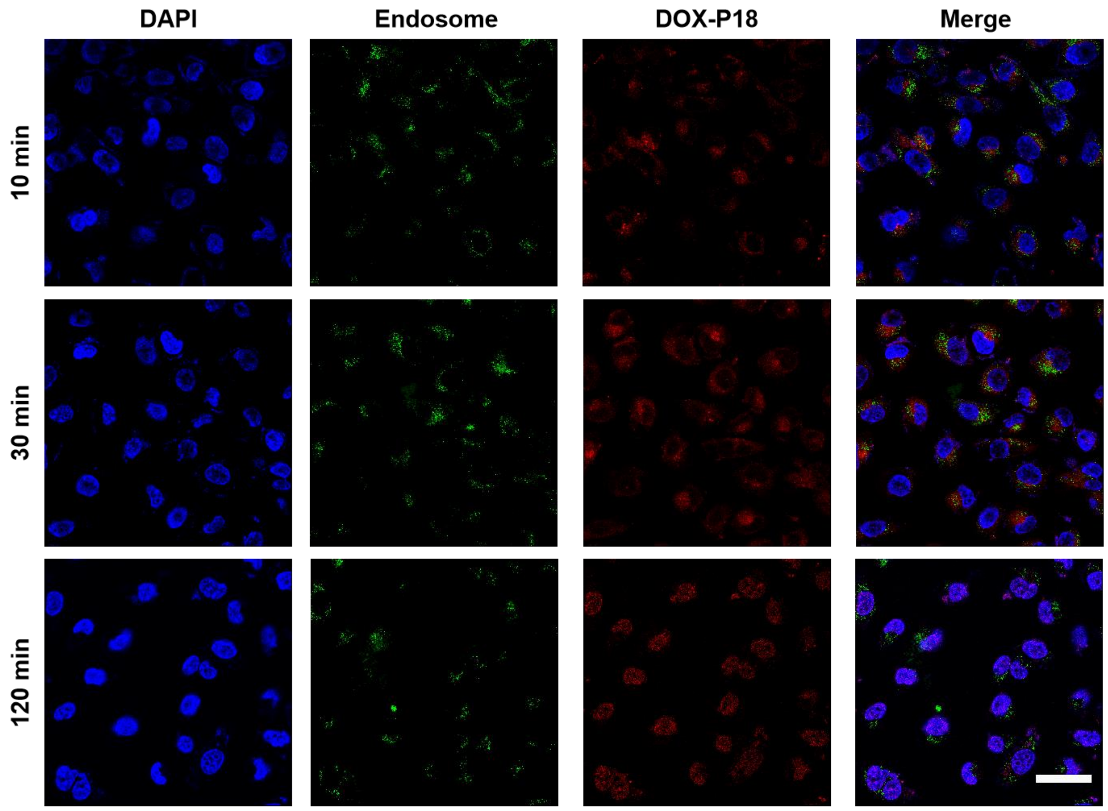

Figure S11. Colocalization of DOX-P18 (red) and DAPI (blue) or endosome tracker green (green) after 10, 30, and 120 min cultured with MDA-MB-231 cells. Scale bar: 25 μm.

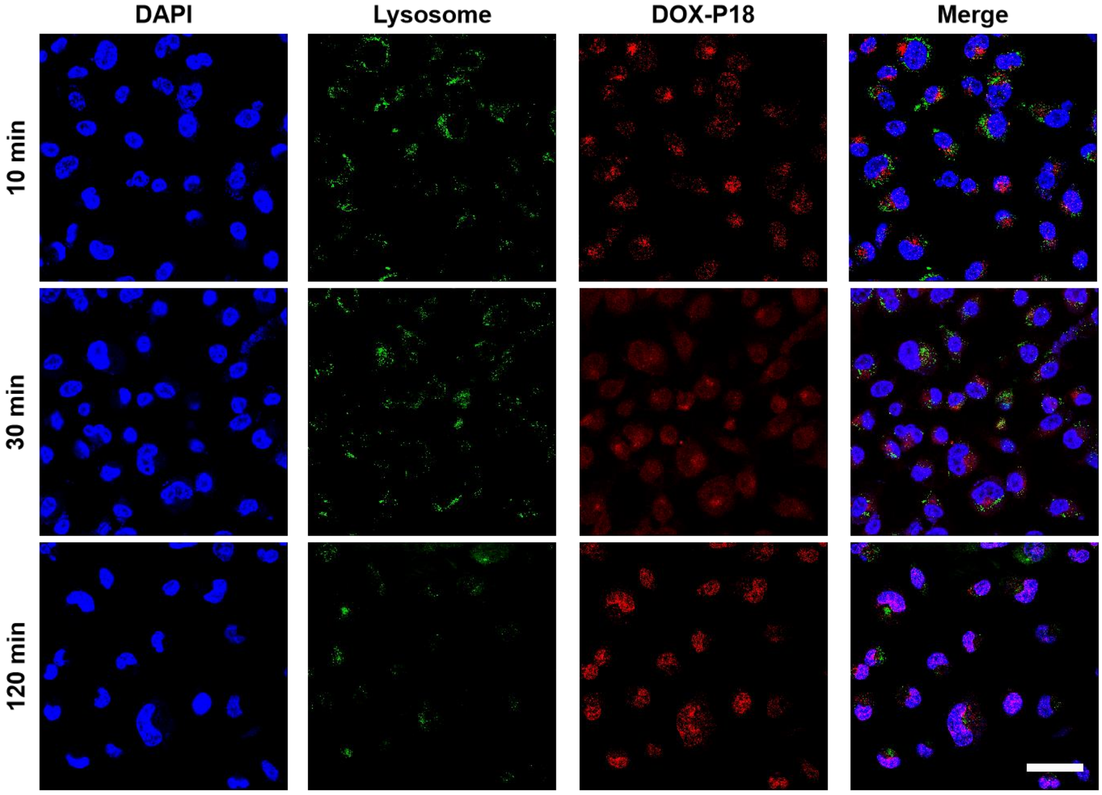

Figure S12. Colocalization of DOX-P18 (red) and DAPI (blue) or lysosome tracker green (green) after 10, 30, and 120 min cultured with MDA-MB-231 cells. Scale bar: 25 μm.

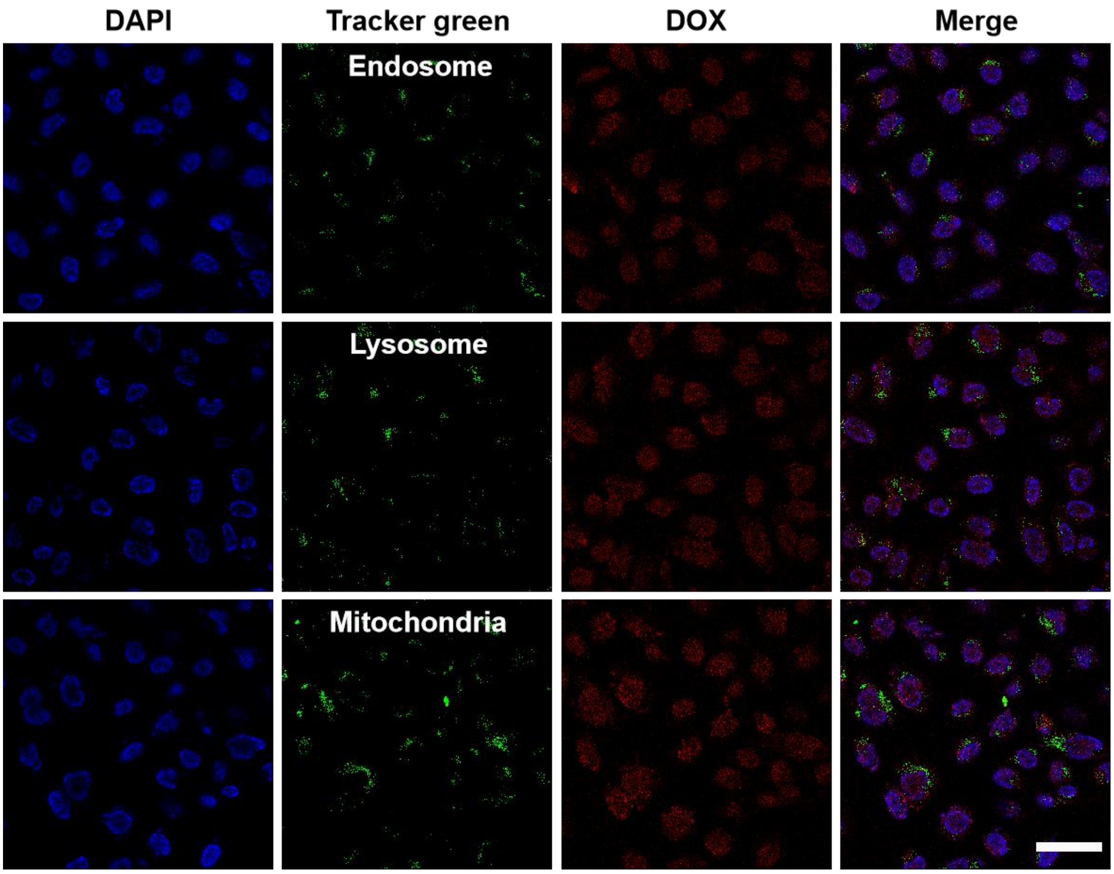

Figure S13. Colocalization of DOX (red) and DAPI (blue) or lysosome/endosome/ mitochondria tracker green (green) after 30 min cultured with MDA-MB-231 cells. Scale bar: 25 μm.

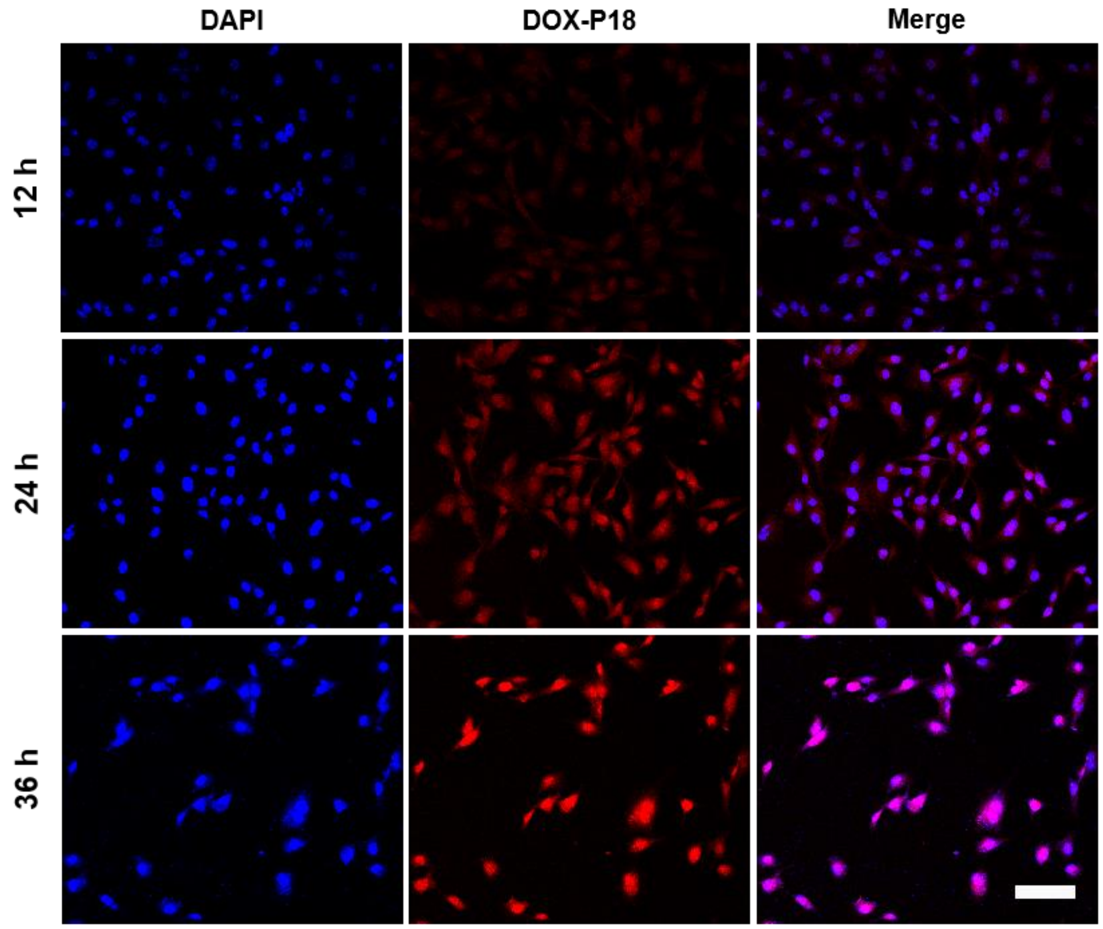

Figure S14. CLSM images of MDA-MB-231 cells incubated with 5 μg/mL DOX-P18 for 12, 24, and 36 h. Scale bar: 100 μm.

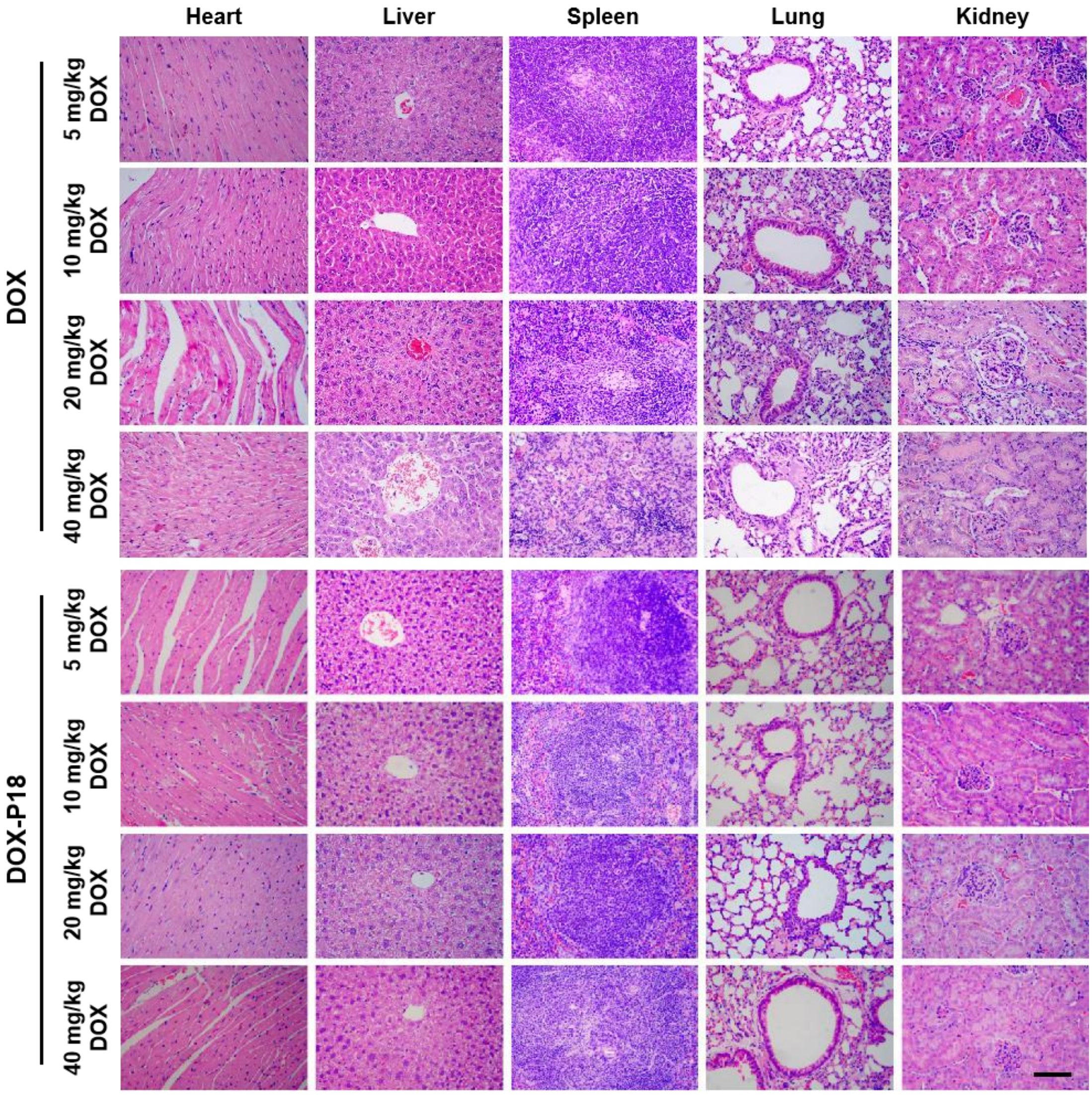

Figure S15. Histological analysis of mice major organs 14 days after injection of single-dose DOX and DOX-P18 with different doses (5, 10, 20, and 40 mg/kg DOX). Scale bar = 200 μm.

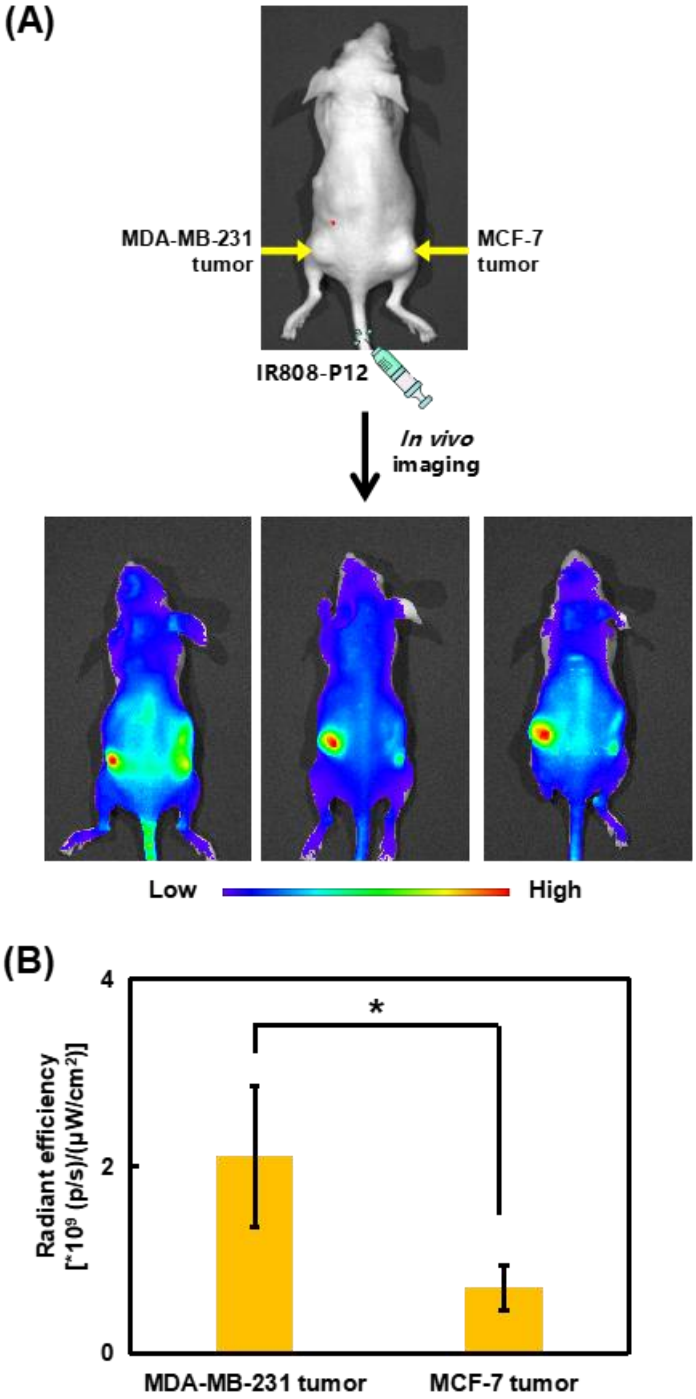

Figure S16. *In vivo* tumor targeting study of NPY analogues P12 on MDA-MB-231 tumor and MCF-7 tumor. (A and B) *In vivo* fluorescence images (Radiance unit: p/sec/cm^2/sr) and quantitative analysis of MDA-MB-231 (left side) and MCF-7 (right side) tumor-bearing mice after intravenous injection of IR808-labled P12 (IR808-P12) at 12 hours. * indicates $p < 0.05$.

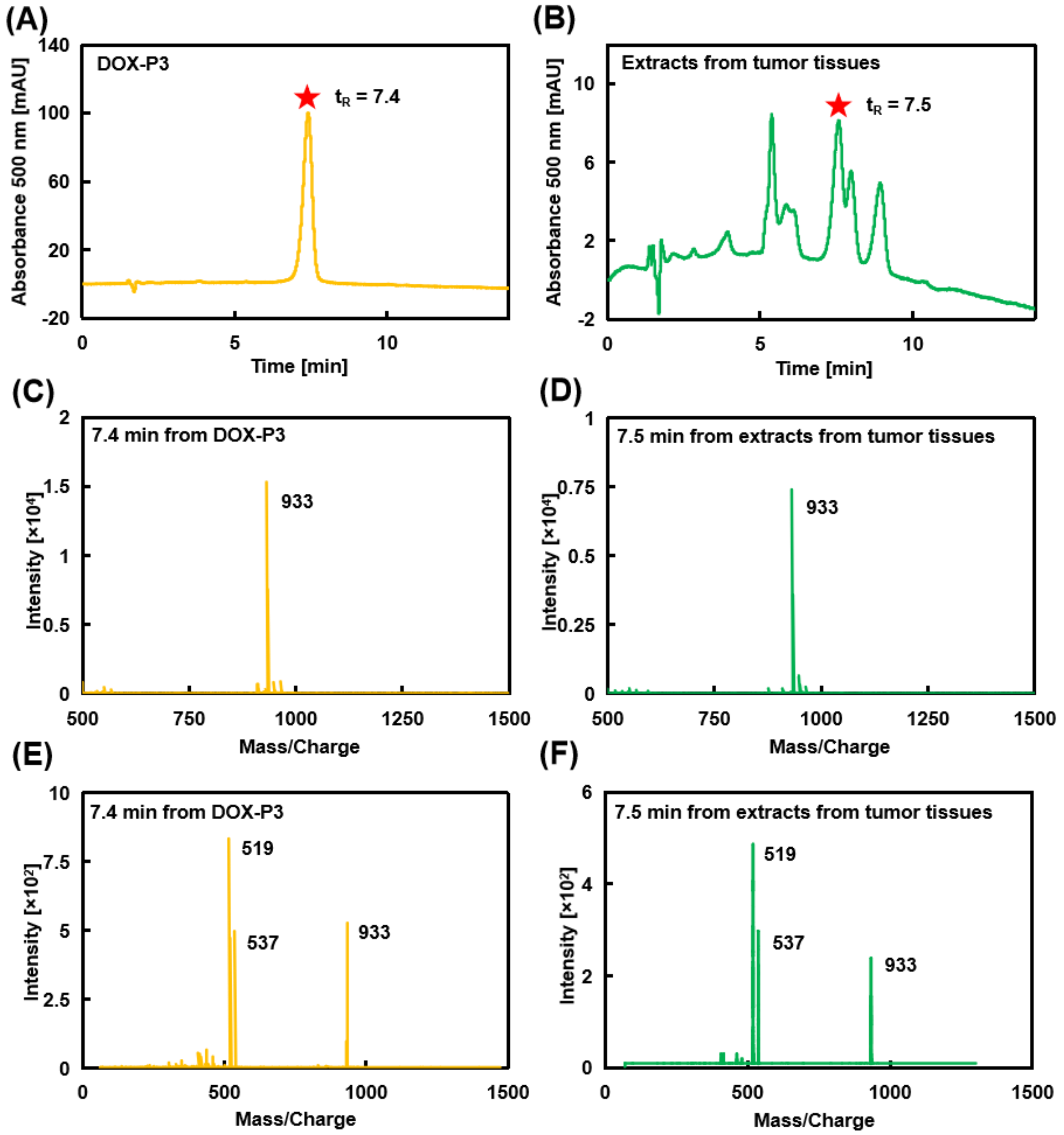

Figure S17. Assessment on MMPs-responsive cleavage of DOX-P18 in situ by LC-MS. (A and B) HPLC analysis of DOX-P3 and extracts from tumor tissues after 2 h intratumoral administration. (C and D) ESI (+) mass spectra of DOX-P3 at 7.4 min and extracts from tumor tissues at 7.5 min. (E and F) ESI-MS/MS spectra of DOX-P3 at 7.4 min and extracts from tumor tissues at 7.5 min.

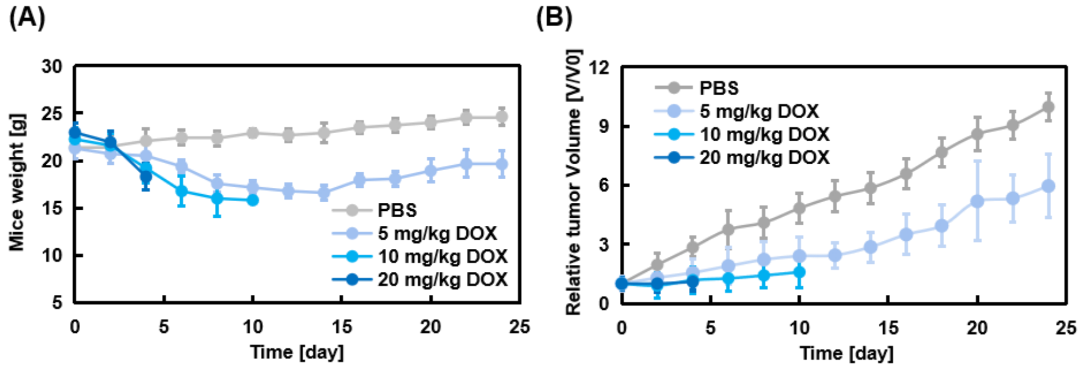

Figure S18. Antitumor efficacy assays of DOX on MDA-MB-231 tumor-bearing nude mice. (A and B) The body weight and tumor volume of mice treated with DOX of different doses. Data are presented as means ± standard deviations (n = 5).

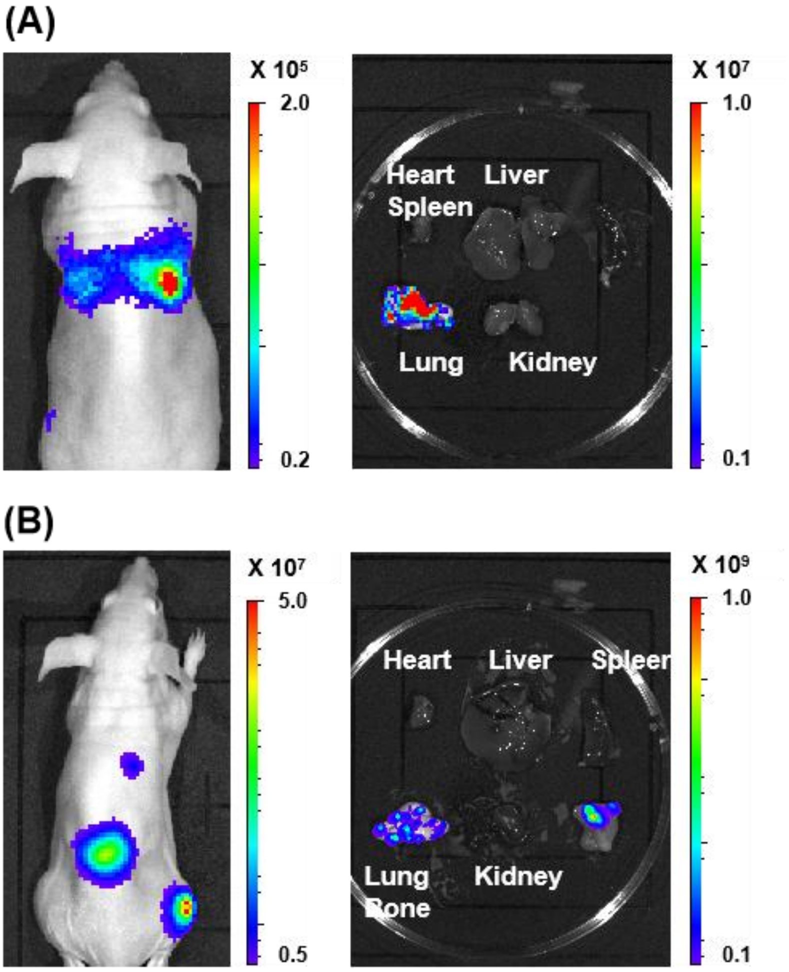

Figure S19. Metastasis models of breast cancer. (A and B) *In vivo* and *ex vivo* fluorescence images (Radiance unit: p/sec/cm^2/sr) of mice at day 12 (D12) and day 26 (D26) after tumor cells MDA-MB-231 injection.

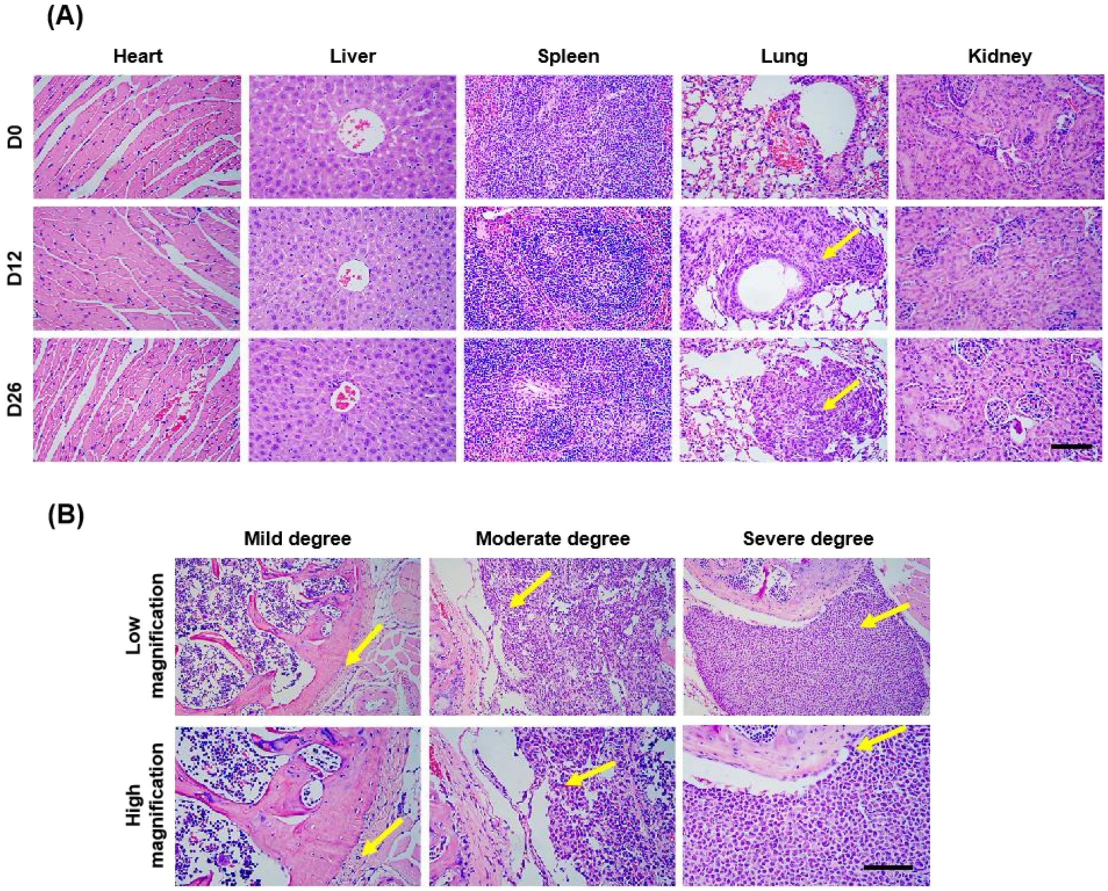

Figure S20. Histological analysis on metastasis models of breast cancer. (A) Histological analysis of mice major organs after intravenous injection of MDA-MB-231 cells at D0, D12, and D26 (400×). (B) Histological analysis of bone tissues at D26 after intravenous injection of MDA-MB-231 cells. Yellow arrows indicate tumor cells in tissues. Scale bar = 200 μm.

Table S1. Hydrated particle size of DOX-P18 in PBS buffer with different pH (7.4, 6.5, 6.0, and 5.0)

	DOX-P18 in PBS pH 7.4	DOX-P18 in PBS pH 6.5	DOX-P18 in PBS pH 6.0	DOX-P18 in PBS pH 5.0
Size [nm]	133 ± 19	ND	ND	ND

Table S2. Mean IC_{50} values for cytotoxicity of DOX, DOX-P18R, DOX-P18, and DOX-P3 on different cell lines after 24 h incubation

	IC_{50} [μg/mL]			
	DOX	DOX-P18R	DOX-P18	DOX-P3
MCF-10A	0.81	1098.8	51.3	12.4
MCF-7	2.2	7388.1	117.5	3.3
MDA-MB-231	0.6	1347.7	7.6	5.6

Table S3. Mean IC_{50} values for cytotoxicity of DOX-P18, DOX-P3, and free DOX on MDA-MB-231 cells after 24, 36, and 48 h incubation

	**IC_{50} [µg/mL]**		
	DOX	DOX-P18	DOX-P3
24 h	1	7.6	5.6
36 h	ND	5.4	4.9
48 h	ND	3.6	2.8

Table S4. MTD of DOX and DOX-P18 in ICR mice (n =10, Male: Female = 1:1)

Drug name	Drug dose [mg/kg DOX]	Number of mice survived	LD_{50} [mg/kg DOX]
Control		10/10	
DOX	5	10/10	26.4
	10	10/10	
	20	8/10	
	40	1/10	
DOX-P18	10	10/10	44.3
	20	9/10	
	40	8/10	
	80	0/10	

Table S5. Pharmacokinetic parameters of DOX and DOX-P18 in plasma

Pharmacokinetic parameters	DOX	DOX-P18
AUC (h·μg/mL)	3.71	15.82
$t_{1/2\alpha}$ (h)	0.09	0.41
$t_{1/2\beta}$ (h)	14.86	10.52
V_{ss} (mL)	231	12.5
CL (mL/h)	26.95	6.32
